# Switchable molecular tweezers: design and applications

**DOI:** 10.3762/bjoc.20.45

**Published:** 2024-03-01

**Authors:** Pablo Msellem, Maksym Dekthiarenko, Nihal Hadj Seyd, Guillaume Vives

**Affiliations:** 1 Sorbonne Université, UMR CNRS 8232, Institut Parisien de Chimie Moléculaire, 4 place Jussieu, 75005, Paris, France

**Keywords:** coordination, molecular recognition, molecular switches, photoswitch, redox, supramolecular chemistry

## Abstract

Switchable molecular tweezers are a unique class of molecular switches that, like their macroscopic analogs, exhibit mechanical motion between an open and closed conformation in response to stimuli. Such systems constitute an essential component of artificial molecular machines. This review will present selected examples of switchable molecular tweezers and their potential applications. The first part will be devoted to chemically responsive tweezers, including stimuli such as pH, metal coordination, and anion binding. Then, redox-active and photochemical tweezers will be presented.

## Introduction

**Figure 1:**
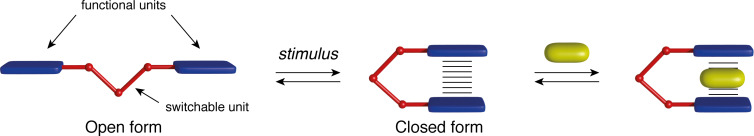
Principle of switchable molecular tweezers.

The terminology “molecular tweezers” was first introduced by Chen and Whitlock in 1978 through a seminal paper [1], wherein they presented a water-soluble molecular receptor composed of two caffeine recognition units linked by a semi-rigid diyne spacer. This ingenious design enabled the tweezers to selectively bind aromatic guests within a cavity, utilizing an induced fit mechanism. Subsequently, with the emergence and advancement of supramolecular chemistry, the field of molecular tweezers experienced rapid expansion, witnessing the development of rigid clips by Klärner [2] and Schrader [3–4], and more flexible variants by Rebek [5–6], Zimmerman [7–8], Bosnich [9] and others [10–12]. Initially serving as agents for guest binding and recognition, molecular tweezers have undergone a remarkable evolution, diversifying their applications into the realms of biology, catalysis, and molecular machines. In particular, the advent of artificial molecular machines [13–14], consisting of an assembly of molecular components that perform mechanical-like motions in response to specific stimuli, has inspired the development of stimuli-responsive molecular tweezers, which have flourished since the early 2000s. It is worth mentioning, as stated by Leigh in a comprehensive review [15], that a pioneering example of a molecular machine was the photoswitchable molecular tweezers developed by Shinkai [16] in 1981 for photocontrolled cation binding. This novel class of tweezers represents prototypes of molecular machines in which guest binding is regulated by stimuli-induced conformational changes between open and closed states (Figure 1). This feature holds significant promise for applications in sensing, drug delivery, or membrane transport within biological systems. Moreover, the incorporation of responsive functionalities into molecular tweezers not only provides significant benefits in catalysis for the development of switchable catalysts but also extends their utility to molecular magnetism, where magnetic switches exploit mechanical motion, and to molecular electronics, enabling multilevel switches.

The stimuli applied to trigger these conformational changes can be categorized into three principal types: chemical, photochemical, and electrochemical. Chemical stimuli involve the introduction of small reactive molecules, such as reagents, ions, or pH changes, and are frequently employed in biological systems for communication and actuation (e.g., ATP). They, however, generate waste which is very well managed in biological systems by using compartmentalization strategies. Nonetheless, the challenge of waste management in artificial systems remains significant. On the other hand, photochemical stimuli offer cleaner alternatives, relying solely on a light source that can be focused on specific areas. However, they may be limited by photostationary states and penetration depth in absorbing media that might prevent full conversion and potential side photochemical reactions. Electrochemical stimuli, while generating no waste when employing electrodes, may face limitations due to the electroactive window of the solvent and require the incorporation of redox-active switching units, which can impose constraints on design and functionality. It is worth noting that the stimulus may affect either the spacer or the functional units, with the former being more commonly employed for the design of switchable molecular tweezers. In this review, we aim to present recent developments in switchable molecular tweezers, classified according to their principal type of stimulus, and explore their applications in various domains.

### Switchable molecular tweezers as an emerging direction in supramolecular chemistry

Among the large variety of molecular machines capable of controlling mechanical motion in response to stimuli, we found switchable molecular tweezers particularly appealing because of the simple design and high potential for many applications. The drastic conformational change induced by the opening and closing of the tweezers attracted us as a straightforward method to control various properties. This change creates a cavity for guest binding, but tweezers have even greater potential if the arms exhibit additional properties such as luminescence, magnetism, catalysis, redox activity, or more. Such systems can also provide two orthogonal responses: the mechanical motion between the open and closed forms, and a potential new property that emerges when the arms are in spatial proximity, either by direct interaction or via a small intercalating molecule. Therefore, switchable molecular tweezers can be considered a prototype of a mechanical molecular device capable of allosteric regulation and dual control through switching and guest binding. While the primary application of molecular tweezers has been in molecular recognition with reversible guest binding, we believe that the scope of applications can be significantly broader. Recent examples have demonstrated their potential to regulate biological activity, facilitate transmembrane transport, enable switchable catalysis, influence magnetic interactions, or serve as multilevel switches. These new directions will be highlighted in the following.

## Review

### Coordination-responsive tweezers

Coordination-responsive switchable systems hold great potential thanks to the tunability and dynamic nature of the coordination bond. This is particularly evident for metal complexes, where the system's geometry can be finely tuned to modulate its response based on the selection of metal and ligand components. This aspect has been extensively explored by Schmittel and co-workers in a recent review [17]. Molecular tweezers have been developed in this direction since the beginning of the 21st century concomitantly with the growth of the field [11,18]. Because of their ability to respond to chemical stimuli, coordination-responsive switchable tweezers have the potential to be used in many applications including but not limited to supramolecular sensors and drug delivery systems [12].

#### pH-Responsive molecular tweezers

A particular case of coordination-responsive systems is when a proton is used as a stimulus leading to pH-responsive systems with the protonation/deprotonation of the switchable moiety. The conformational switch in these systems is mainly driven by intramolecular hydrogen bonds.

**Figure 2:**
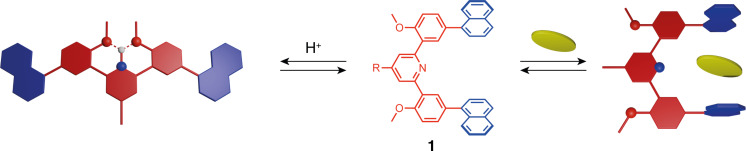
Principle of pH-switchable molecular tweezers **1** [19].

The methoxyphenyl-pyridine-methoxyphenyl moiety, developed by Petitjean et al. [19], demonstrates conformational switching upon the addition of acid in aqueous media (Figure 2). The neutral tweezers adopt a "U"-shaped conformation with both arms pointing in a parallel direction, as the bulky -OMe groups are located outside the tweezer’s cavity to avoid steric hindrance with each other. When protonated, the pyridinium group acts as a hydrogen-bond donor for the two methoxy groups and triggers the rotation of the respective benzene rings along with the attached functional arms. The formation of new hydrogen bonds between -OMe groups and protonated pyridine stabilizes a "W"-shaped open conformation. The conformation switch can be easily followed by NMR spectroscopy in solution. Remarkably, the water-soluble tweezers **1**, when functionalized with hydrophobic arms, can encapsulate planar aromatic molecules, making this system promising for drug delivery.

**Figure 3:**
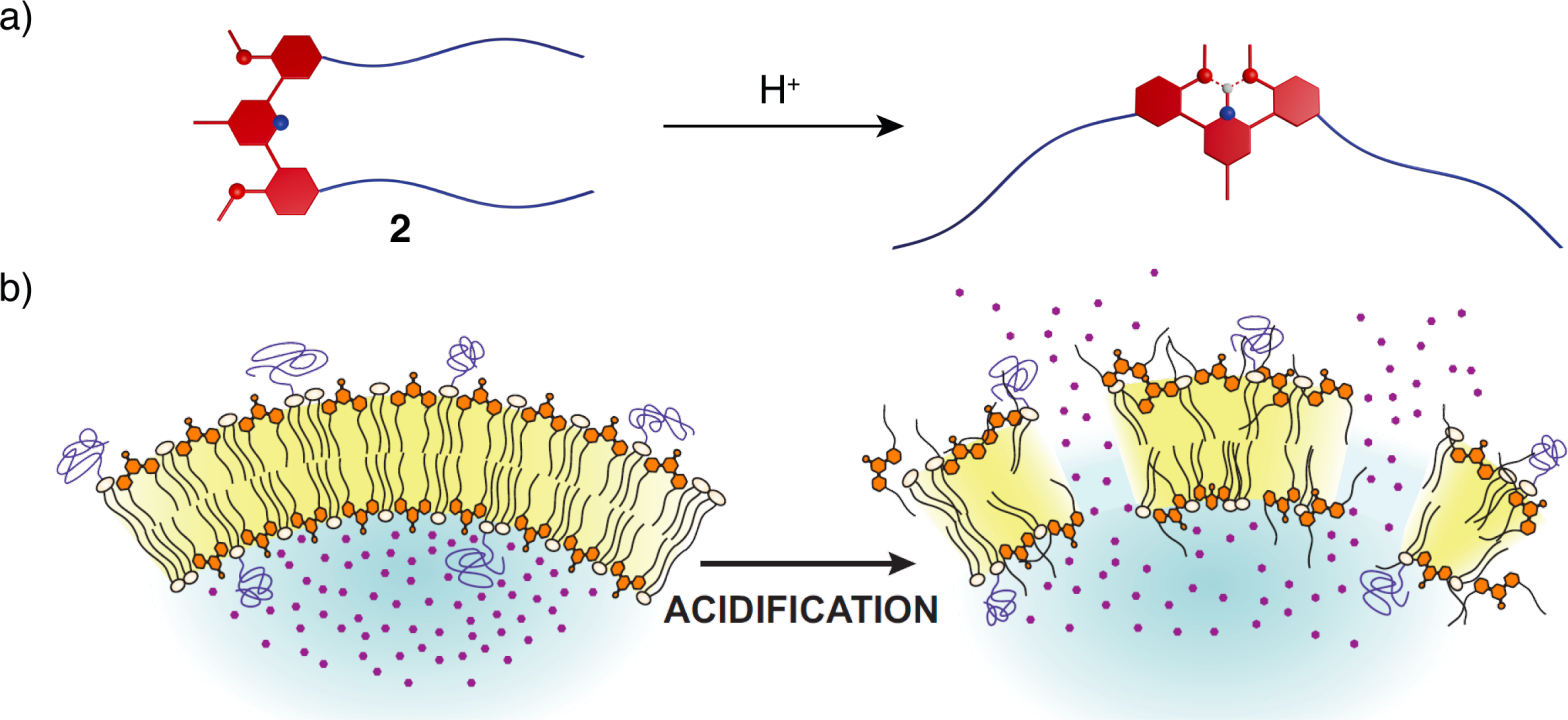
a) pH-Switchable tweezers **2** substituted with alkyl chains as switchable lipids. b) Schematic depiction of the lipid bilayer disruption mechanism induced by **2**. Figure 3b was reproduced from [20] W. Viricel et al., “Switchable Lipids: Conformational Change for Fast pH-Triggered Cytoplasmic Delivery” Angew. Chem., Int Ed. with permission from John Wiley and Sons. © 2015 WILEY-VCH Verlag GmbH & Co. KGaA, Weinheim. This content is not subject to CC BY 4.0.

The advancement of pH-switchable molecular tweezers laid the groundwork for the development of switchable lipids [20]. When such lipids are incorporated in lipid vesicles, they provide means for controlled release of siRNA or miRNA encapsulated within them into the solution in vitro and in vivo [21]. The closed conformation of **2** with parallel alkyl chains acts as a building block of the bilayer membrane and is packed together with other lipids. When the surrounding medium becomes acidic, the tweezers adopt their open conformation. This causes fluctuations and local defects in the packing of a bilayer (Figure 3), leading to membrane disruption and release of active components from within the vesicles [22]. Such concept makes these tweezers good candidates for controlled drug delivery.

**Figure 4:**
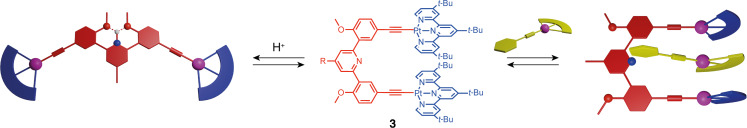
Modification of spectral properties of **3** by controlled induction of Pt–Pt interactions.

The group of F. Wang et al. exploited the same scaffold to control the physical properties of materials. They incorporated planar terpyridine-alkyne Pt complexes as functional units and studied the intercalation of a Pt-complex guest in order to obtain Pt–Pt interactions in solution [23]. In the native state of **3**, both arms with planar Pt complexes are parallel and the distance between them allows for the intercalation of another terpy-Pt complex. The guest intercalation coupled with the induction of short-range Pt–Pt interactions was followed by UV–vis absorption and emission spectroscopies with characteristic MMLCT signals in the low-range visible/NIR region. Upon protonation of the pyridine, the conformation switch leads to a spatial separation of the active Pt moieties and a release of the guest (Figure 4). Also, the same group demonstrated the induction of chirality and fluorescence with chiral guest molecules using a similar principle [24]. The protection from the solvent of the intercalated Pt guest enables its fluorescence emission and is accompanied by the induction of chirality in the resulting host–guest complex. A significant enhancement of the circular dichroism response of the chiral guest is observed confirming the formation of the host–guest complex. Again, protonation of the pyridine results in a guest release and a loss of the CD signal. Thus, this system provides a peculiar example of the control of chirality by a pH stimulus.

**Figure 5:**
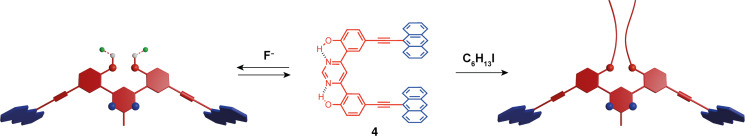
Conformational switching of di(hydroxyphenyl)pyrimidine-based tweezer **4** upon alkylation or fluoride anion addition.

A similar moiety employed for acid–base-triggered conformational switching is di(hydroxyphenyl)pyrimidine which was developed by Osakada et al. [25] (Figure 5). By default, this moiety adopts a U-shaped conformation stabilized by OH···N hydrogen bonds. In this conformation, tweezers **4** with two 9-ethynylanthryl arms form a 1:1 complex with 2,4,7-trinitrofluorenone (TNF) with a good association constant of 2100 M^−1^. Upon protonation of the pyrimidine nitrogen atom the hydrogen bond is disturbed, which should result in a conformational change to an open W-shaped form. Even though an acid-mediated hydrogen bond disruption was expected, no clear experimental evidence was reported. Nevertheless, conformational changes were obtained from two other stimuli: (i) alkylation of phenolic OH groups [25] that leads to the disappearance of the hydrogen bonds and a more stable W-shaped conformation; (ii) addition of F^−^ anion [26] as a competitive hydrogen-bond acceptor that binds to the OH groups instead of the pyrimidine nitrogen atoms.

**Figure 6:**
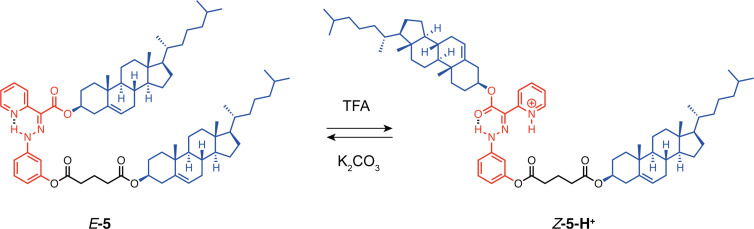
Hydrazone-based pH-responsive tweezers **5** for mesogenic modulation.

The group of Aprahamian has developed hydrazone-based molecular switches that can be controlled by photochemical or chemical stimuli such as pH [27]. They reported mesogenic tweezers-like compound **5** composed of a hydrazone switch substituted by two mesogenic cholesteryl groups (Figure 6) [28]. Due to strong hydrogen bonding between the pyridyl moiety and the N–H of the hydrazone, tweezers **5** predominantly exist in the closed *E*-form in a CD_2_Cl_2_ solution (*E*/*Z*-isomer ratio of 91:9). Upon protonation of the pyridyl group, a complete conversion to the *Z* open form is achieved, in which the cholesteryl units are oriented in an *anti*-fashion. This conformational change results in a significant increase in the solvent-accessible surface area. Tweezers **5** were utilized as a dopant for the achiral liquid crystalline material nematic phase 5 (NP5) to produce a chiral nematic phase, whose reflected color can change from green to purple under cross-polarized view upon protonation. This system serves as an elegant example of a macroscopic effect induced by a conformational change at the molecular level.

**Figure 7:**
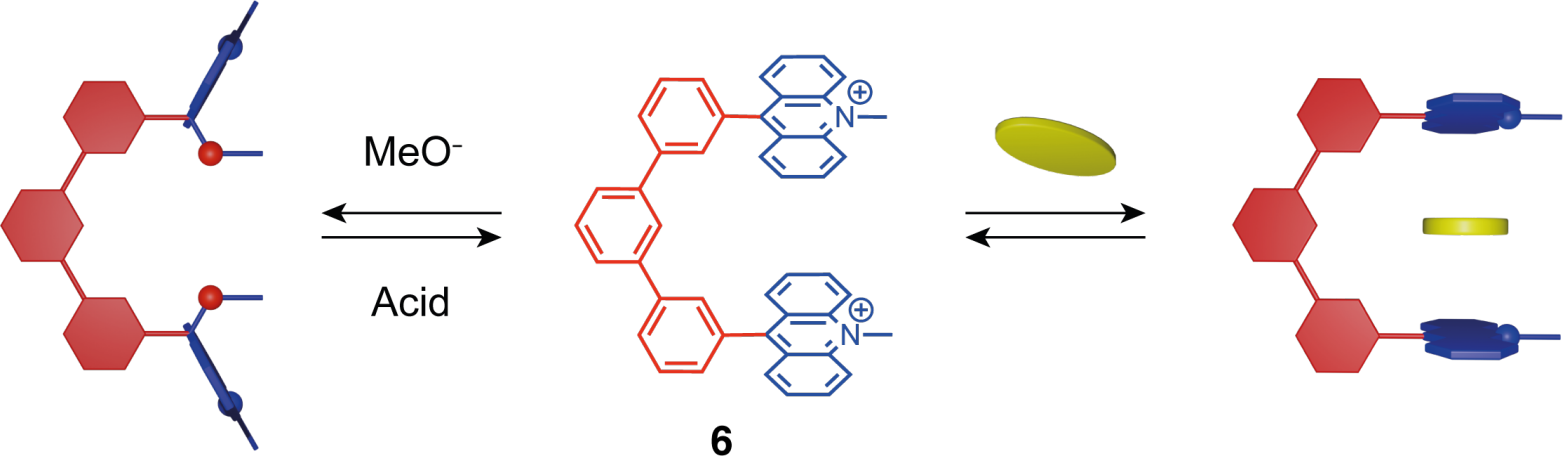
pH-Switchable molecular tweezers **6** bearing acridinium moieties.

In a different approach where the stimuli affect the recognition unit, pH-switchable molecular tweezers with acridinium functional units have been recently reported by de Rouville et al. [29]. The acridinium moiety is a planar electron-poor aromatic system that can form π–π interactions with electron-rich molecules [30]. The particularity of acridinium is that it can undergo the addition of a nucleophile like HO^−^ or RO^−^ forming an acridane derivative having non-planar geometry and different electronic properties. Triphenylene tweezers **6** are able to intercalate planar electron-rich guest molecules like TTF and pyrene. Upon the addition of a nucleophile (MeO^−^) the acridinium moieties are modified to acridane which leads to the loss of affinity for the guests (Figure 7). This process is reversible as the addition of an acid restores the acridinium planar form. Thus, such molecular tweezers are also good candidates for controlled guest release in solution. This concept was further developed with a bis-acridinium cyclophane [31] as a multiresponsive receptor for selective phase transfer. In organic media, this macrocyclic receptor presented an affinity for polyaromatic guests with strong selectivity for perylene. A reversible guest release was achieved by chemical (hydroxide/proton) or electrochemical (reduction/oxidation) stimuli allowing the enrichment of perylene from a mixture of polycyclic aromatic hydrocarbons (PAHs) in phase-transfer experiments into a perfluorocarbon phase.

### Metal cation responsive tweezers

**Figure 8:**
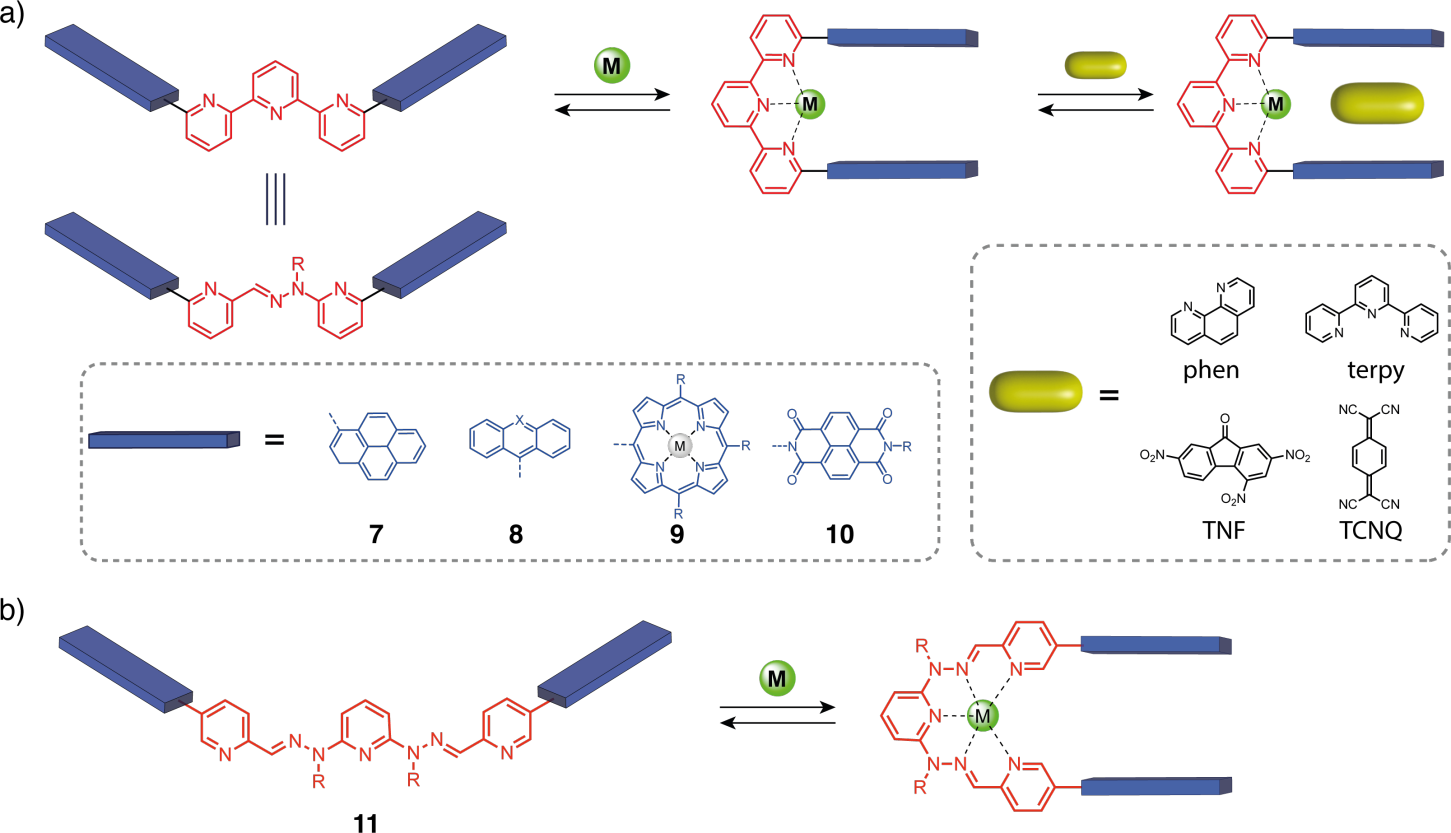
a) Terpyridine and pyridine-hydrazone-pyridine analogs molecular tweezers and b) extended pyridine bishydrazone tweezers for guest binding.

In the early 2000s, Lehn and co-workers introduced a switchable system based on a terpyridine (terpy) ligand (Figure 8), which is structurally similar to the diphenylpyridine units used by Zimmerman in rigid clips, but can change their conformation upon complexation by a metal cation [32–33]. When substituted at the 6 and 6" positions, the terpyridine adopts an open "W"-shaped conformation due to the s-*trans*-conformation between two pyridines forced by the repulsion of the nitrogen lone pairs. Upon metal coordination the pyridine units rotate to allow for a tridentate binding to the metal cation, thus inducing a molecular motion to a closed “U”-shaped conformation. This type of switch was first described with the molecular tweezers **7** bearing two pyrene chromophores on the position 6 and 6” of the terpyridine, resulting in an open “W” conformation [33–34]. In this state, the system exhibits luminescence properties that are quenched when the tweezers are closed by the addition of a Zn^2+^ cation because of intramolecular π–π stacking interactions between the chromophores. The system can be reopened and its luminescence properties restored by introducing tris(2-aminoethyl)amine (TREN), which has a better affinity for Zn^2+^ and can abduct it from the terpyridine. The luminescence properties in the closed state can also be modulated by the formation of host–guest complexes. This happens with ligands complexing the metal center only, such as terpyridines and phenanthrolines. In fact, the counterions of the zinc^2+^ cation and their solvation sphere occupy part of the cavity and prevent non-coordinating guests from entering the binding cavity. By extending the spacer between the terpy and the arms from a single C–C bond to an amide functional group that also participates in the coordination of the Zn^2+^, non-coordinating guests such as trinitrofluorene (TNF) and tetracyanoquinodimethane (TCNQ) are complexed in the closed state [32].

Terpyridine-based switches have also been described with metalloporphyrin arms bearing metal ions such as Zn(II) and Au(III) [35]. Terpyridine bisporphyrin tweezers have been synthesized as homometallic (Zn/Zn) and heterometallic (Zn/Au) compounds. In the open state, the porphyrins do not interact and the closing process allows the system to acquire distinct properties depending on the porphyrin. For the Zn/Zn system, guest binding abilities have been observed with diamines that can coordinate the two metallic centers in the tweezers cavity in a bridging mode. For the heterometallic Zn/Au system, luminescence quenching was observed in the closed state.

The pyridine-hydrazine-pyridine unit has been extensively studied by Lehn and co-workers in coordination-responsive supramolecular polymers [36] as it is isomorphic to the terpyridine and presents similar metal coordination properties [36–38]. Molecular tweezers **10** bearing 1,4,5,8-naphthalenediimide (NDI) luminescent arms have thus been developed. The system exhibits similar switching behavior as the terpyridine tweezers. In terms of host–guest complexation, it can also only bind to coordinating guests (terpyridine, bipyridine) due to the proximity of the coordination sphere with the binding pocket. Tweezers **11** with NDI arms and an extended pyridine-hydrazone-pyridine-hydrazone-pyridine switchable unit have thus been developed. This larger system adds two more chelating sites and moves farther the functional unit allowing the intercalation of electron-rich polyaromatic guests such as pyrene [39]. In general, these hydrazone-based systems have the advantage of being more synthetically accessible than the terpyridine-based system and enable dynamic ligand formation due to the reversibility of the hydrazone bond formation [40].

**Figure 9:**
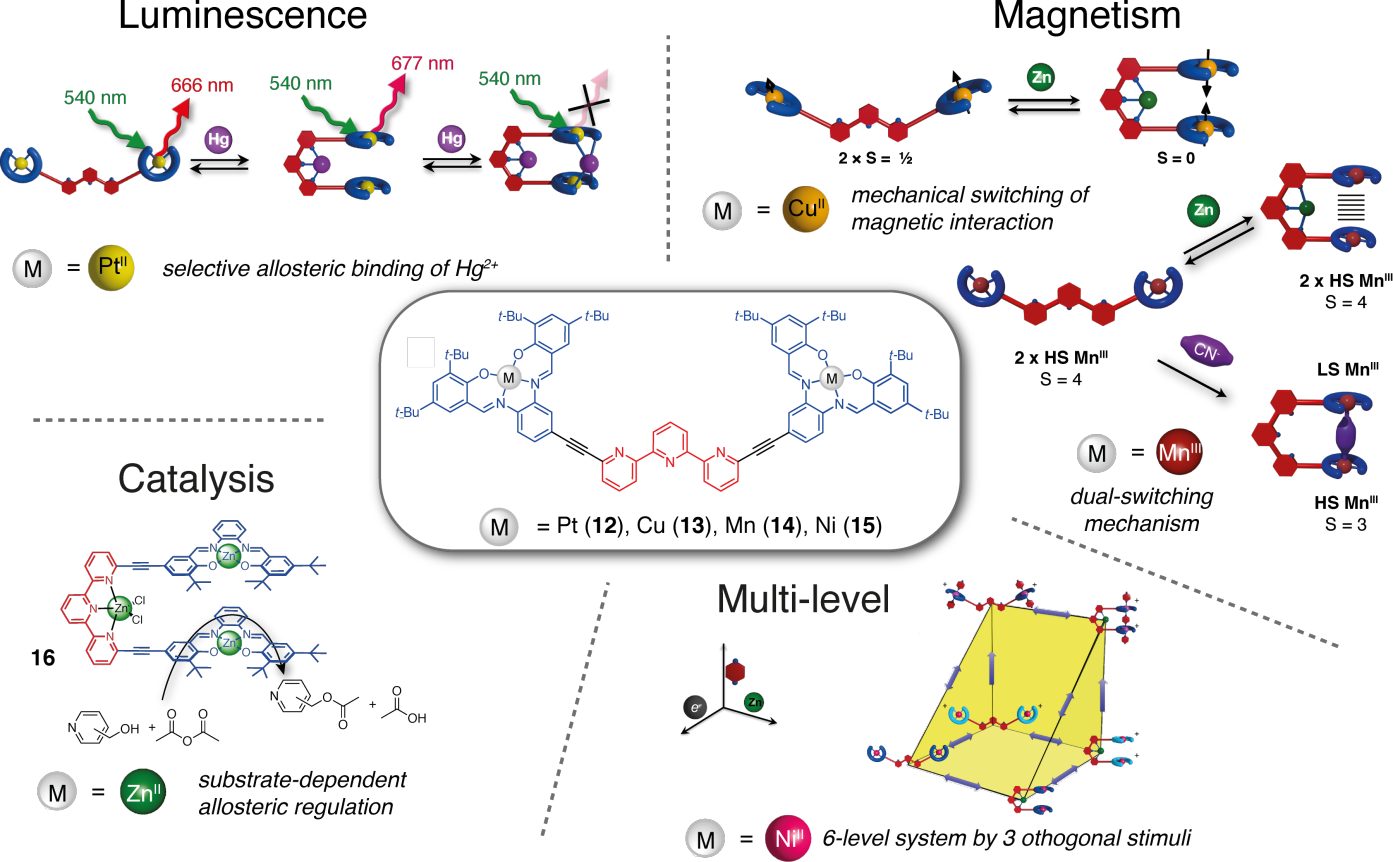
Terpyridine-based molecular tweezers with M–salphen arms and their field of application. Figure 9 was adapted with permission from [46], Copyright © 2017 American Chemical Society. This content is not subject to CC BY 4.0.”

More recently, Vives and co-workers have developed a family of terpyridine-based tweezers bearing metal–salphen complexes as functional units (Figure 9). Using metal complexes as arms brings modularity to the system and allows exploring applications beyond guest recognition for molecular tweezers. Indeed, with the same design of tweezers, their properties can be modified just by changing the complexed metal in the functional unit. Such tweezers were first described with 6,6”-substituted terpyridine-bearing platinum–salphen known for their luminescence properties [41]. The closing process of the tweezers **12** was studied by NMR or UV–vis titrations with Zn^2+^ and other cations such as Pb^2+^, Fe^2+^, Cu^2+^, Eu^3+^, and Yb^3+^ indicating in all cases a 1:1 association model. The system can then be reversibly reopened by the addition of TREN as a competitive ligand. The authors reported a slight luminescence quenching in the zinc-closed state with a decrease in the quantum yield from 0.27 to 0.21. However, Hg^2+^ presented a special behavior with the formation of a 2:1 complex, the closing of the tweezers by the first equivalent generating an allosteric binding site specific to a second Hg^2+^ ion. While the addition of one equivalent of Hg^2+^ decreased the quantum yield to 0.09, the formation of a bis-coordinated [tweezers-Hg_2_]^4+^ complex resulted in a total luminescence quenching (quantum yield <10^−3^). The crystallographic structure of the closed form revealed a helicoidal folding of tweezers resulting in a stacking of the Pt–salphen moieties with a Pt–Pt distance of 3.75 Å slightly above the limit for Pt–Pt interactions. In order to enable better interactions in the closed form, modified Pt–salphen tweezers with *tert*-butyl groups positioned farther from the salphen via alkyne spacers were synthesized [42]. While, like in the parent tweezers **12**, no intercalation of aromatic guests was observed in the closed form, strong intramolecular and intermolecular Pt–Pt bonds were achieved in the solid state.

Using the same architecture, the group explored the control of magnetic properties by the mechanical motion of the tweezers using Cu(II)–salphen complexes [43]. The magnetic properties of tweezers **13** were studied by EPR spectroscopy and SQUID magnetometry. In the open state, the large intramolecular distance of 21 Å between the two Cu(II) complexes results in an independent paramagnetic behavior. However, in the closed form, the metal centers are much closer to each other (4.03 Å) resulting in a weak antiferromagnetic coupling via through-space exchange interaction. This demonstrates the value of coordination-switchable tweezers for switching between two magnetic states that are stable at room temperature [44]. To increase the magnetic coupling between the metal center the group investigated the Mn(III)–salphen tweezers **14** that can coordinate in apical position to cyanide ions. The complexation of CN^−^ anions to the open form unexpectedly resulted in the closing of the tweezers thanks to a Mn–CN–Mn bridging [45]. An antiferromagnetic coupling between the two Mn(III) centers combined with a spin crossover from high spin to low spin Mn(III) was observed. Like in the other terpy-based tweezers, a reversible Zn-induced closing was also achieved. Despite the magnetic anisotropy of Mn(III), no single molecule magnet behavior was observed in either closed form. Nevertheless, this system constitutes a promising example of dual switchable molecular tweezers that can be addressed by two orthogonal stimuli.

Vives and co-workers then explored the redox non-innocent properties of Ni(II)–salphen moieties to achieve a multistate system with tweezers **15** [46]. Indeed, Ni(II)–salphen complexes present two reversible oxidation waves with oxidation centered on the ligand. However, in the presence of a coordinating ligand (such as pyridine or pyrazine), valence tautomerism from ligand-centered oxidation to a metal-centered one is achieved due to the stabilization of an octahedral Ni(III). These properties allow the tweezers to reach six distinct and stable states by playing with three orthogonal stimuli that are: (i) metal coordination for closing/opening of the tweezers, (ii) reversible oxidation, (iii) pyrazine guest binding in the oxidized state. Such an example demonstrates the potential of the molecular tweezers architecture to go beyond simple two-level switches and access multilevel systems. Catalytic tweezers **16** were then synthetized by Vives and co-workers using a 6,6”-substituted terpyridine this time bearing zinc(II)–salphen complexes [47]. The catalytic activity was evaluated in an acetyl transfer reaction between pyridinemethanol derivatives and anhydrides. For the *ortho* derivative, a rate increase was observed upon closing due to the spatial proximity in the cavity between the two substrates. In contrast, the open tweezers showed a higher rate for the acetylation of *meta* and *para* substrates as a result of substrate inhibition of the closed cavity. These tweezers constitute a remarkable substrate-dependent allosteric with potential applications for regulating cooperative catalytic reactions.

**Figure 10:**
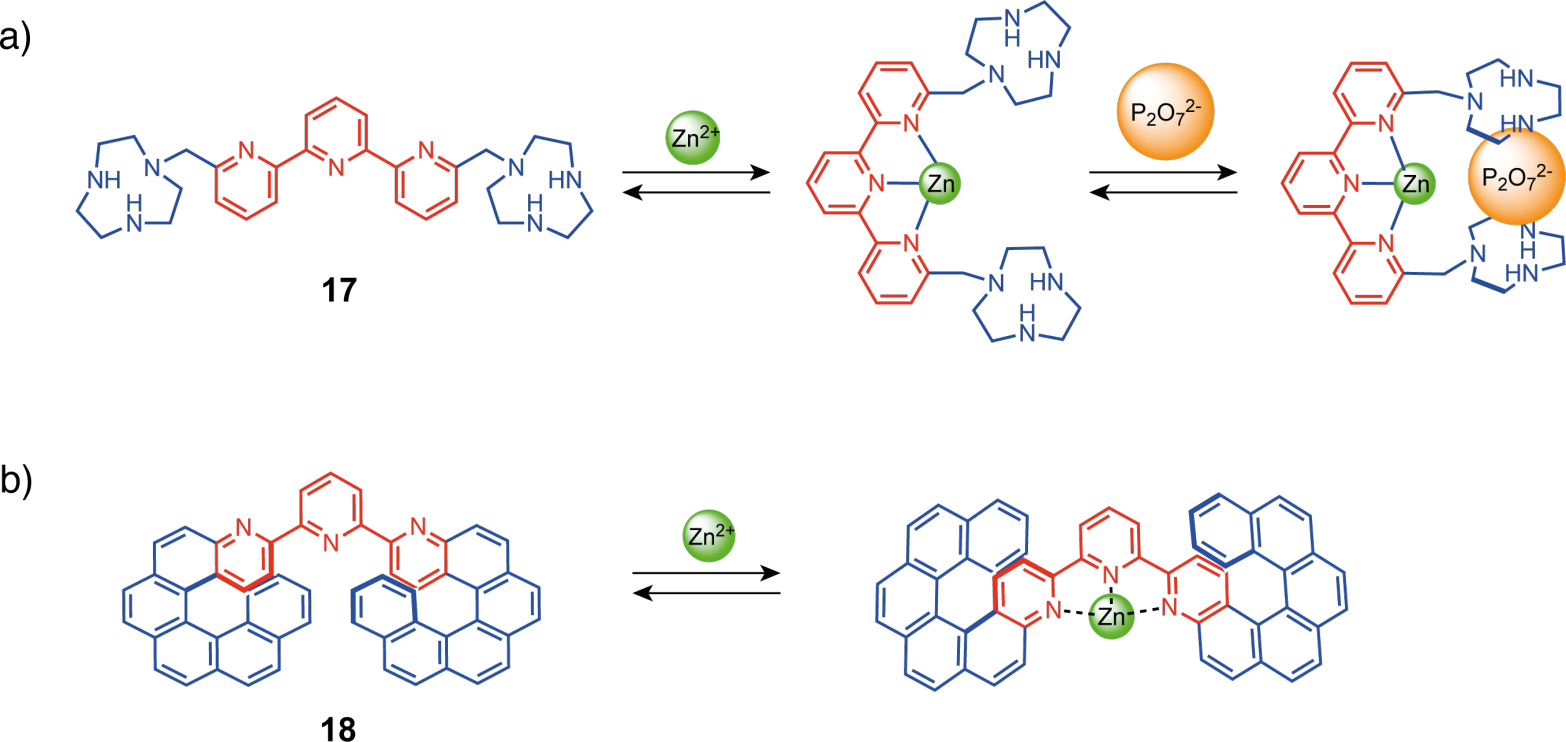
a) Terpyridine-based molecular tweezers for diphosphate recognition [48]; b) bishelicene chiroptical terpyridine-based switch [50].

Terpyridine-based tweezers have also been developed by Bencini, Lippolis, and co-workers for the selective recognition of diphosphate with [9]aneN_3_ unit **17** [48]. When closed with Zn(ClO_4_)_2_, the anion-binding moieties are preorganized for diphosphate binding, resulting in a 3-order of magnitude increase in affinity compared to the open form (log K = 6.9 vs 2.9). This positive allosteric response benefits from ion pairing with the metal cation in addition to the diphosphate interactions via hydrogen bonding with the two protonated [9]aneN_3_ units and two water molecules coordinated to the zinc center. More recently, terpyridine was also used by Crassous and co-workers to create a chiroptic switch (Figure 10) [49]. They synthesized tweezers **18** bearing helicene moieties as functional groups. The two enantiomers (*P*,*P*) and (*M,M*) were separated by chiral HPLC and displayed opposite circularly polarized luminescence (CPL) and electronic circular dichroism (ECD) properties. The addition of ZnCl_2_ switched the system from a compact conformation to an extended conformation, resulting in a modulation of the chiroptical properties with a large change in the absorption spectra and a bathochromic shift in the emission maximum. This system represents a rare example of a multi-output readout system, showing responses in ECD, fluorescence, and CPL activity. The authors later reported a similar system with a bipyridine switching unit that exhibits similar chiroptical switching properties [50].

**Figure 11:**
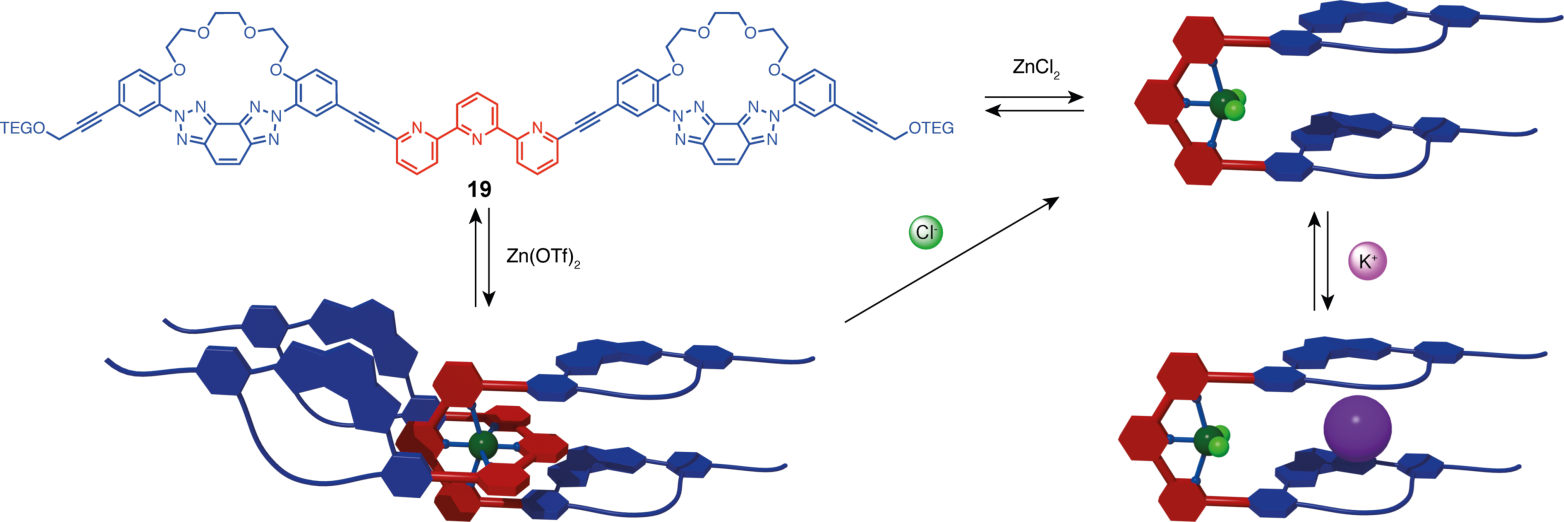
Terpyridine-based molecular tweezers with allosteric cooperative binding.

During the writing of this review, Lee et al. reported molecular tweezers presenting an allosteric response with hard–soft cooperativity [51]. Tweezers **19** is based on a terpyridine ligand substituted in a 6,6” position by azacrown macrocyclic units. As shown previously, the tweezers adopt an open “W” form that can be switched to a closed “U” form by Zn^2+^ complexation. However, in this system, depending on the zinc counter ions either a bis(terpy) 2:1 complex or a 1:1 complex is obtained (Figure 11). With non-coordinating triflate counter ions, the bis(terpy) complex is exclusively formed in CDCl_3_/CD_3_OD 4:1. This complex can be converted to the 1:1 species upon exchange of the triflate with more coordinating chloride anions or directly formed upon closing with ZnCl_2_. Interestingly, only the closed 1:1 form presents a selective allosteric binding to alkali K^+^ cations associated with a shift in the emission maxima. This system offers innovative exploitation of switchable molecular tweezers for allosteric ion recognition with a double selection (metallic ion and counter anion) of the closing stimulus.

**Figure 12:**
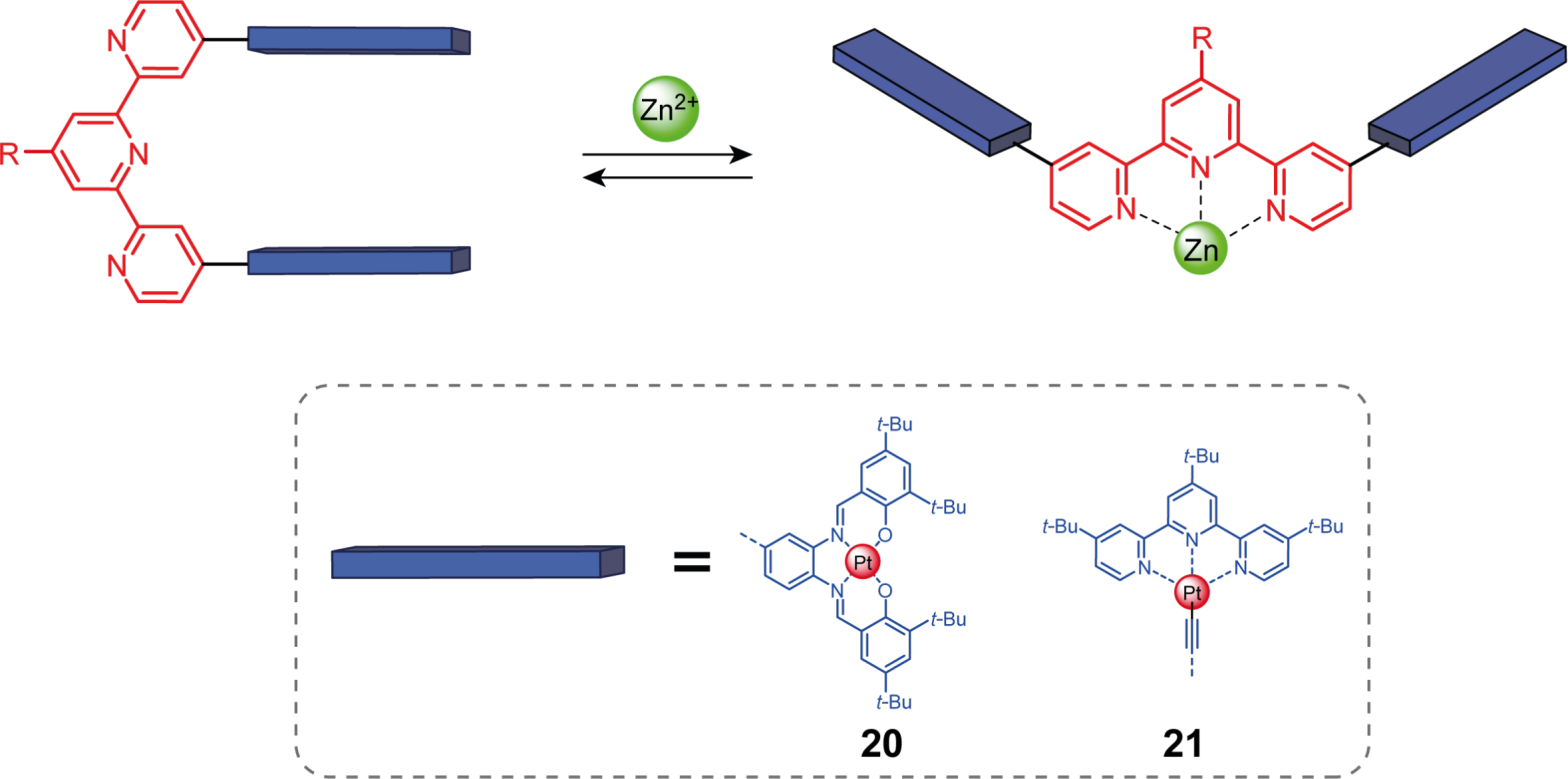
Terpyridine-based molecular tweezers presenting closed by default conformation.

Closed-by-default tweezers can be achieved by changing the substitution pattern of the terpyridine from 6,6” to 4,4” (Figure 12). Due to the repulsion between the nitrogen lone pairs, the 4,4”-substituted terpy adopts a “U” conformation in the non-complexed state and can be switched by metal coordinating to a “W” open conformation. Vives and co-workers reported bis(platinum–salphen)terpyridine tweezers **20** that can intercalate in the U form extended aromatic guests such as coronene and perylene [52]. The opening of the tweezers with the addition of Zn^2+^ released the guest demonstrating an example of negative allosteric regulation with molecular tweezers. It should be noted that the coordination of Zn^2+^ resulted in a 2:1 bis(terpyridine) [Zn(**19**)_2_]^2+^ complex that was not observed in the analog 6,6” terpy-based tweezers **12**.

Wang and co-workers also reported closed-by-default molecular tweezers based on 4,4”-substituted terpyridine bearing this time alkynylplatinum(II)–terpyridine arms **21** [53]. The intercalation of an alkynylplatinum(II)–terpyridine complex system results in a large absorption band in the visible domain (λ_max,abs_ = 515 nm) and an enhanced emission band in the infrared (λ_max,emission_ = 780 nm) attributed to the proximity of the metallic centers which allows MMLCT transitions. These properties have been used to generate reactive oxygen species (ROS) and efficient photocatalytic oxidative cyanation of *N*-phenyl-1,2,3,4-tetrahydroisoquinoline. The photocatalytic activity of the catalyst could be allosterically imbibed by the addition of zinc(II) which opens the tweezers, releasing the guest and leading to the disappearance of the photosensitizing properties of the system. In an extension of this work, discrete tetranuclear Pt complexes with Pt–Pt interaction were obtained by self-assembly between **21** and bisalkynylplatinum(II)–terpyridine clips [54]. The dimer showed photocatalytic activity in the photooxidation of a secondary amine to the corresponding imine that could be deactivated and reactivated by opening or closing the tweezers.

**Figure 13:**
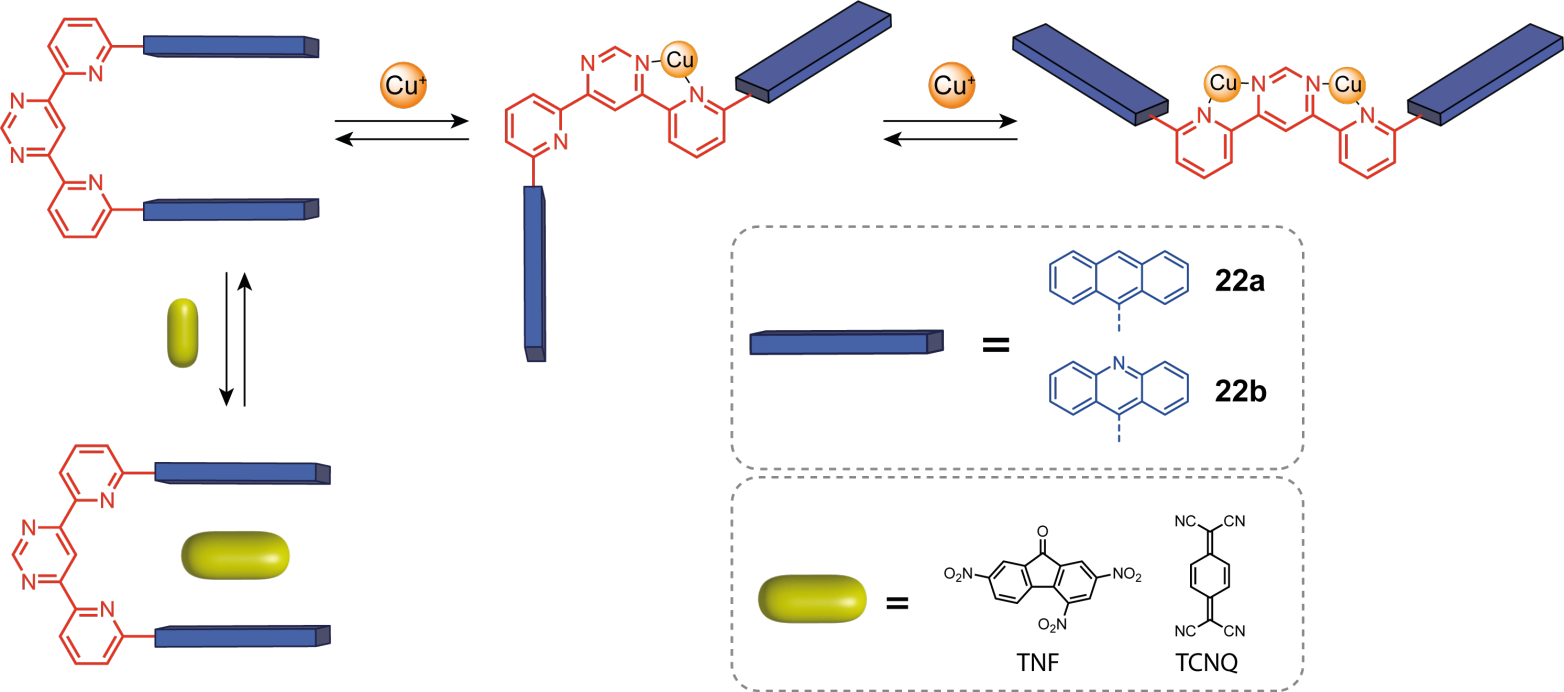
Pyridine-pyrimidine-pyridine-based molecular tweezers.

Variations on multidentate N-donor ligands have also been developed by Lehn and co-workers to introduce new behaviors for cation-responsive systems. One of them is a pyridine-pyrimidine-pyridine (py-pym-py) moiety that, when substituted in 6,6” positions, adopts a “U” conformation due to the lone pairs repulsion between the central pyrimidine and side pyridine nitrogen (Figure 13) [32]. Such moiety is able to complex two copper(I) cations, both sides of the system acting as independent bipyridine units, giving the possibility of sequential opening of the tweezers one arm at a time. Indeed, when only one copper(I) cation is complexed, tweezers **22a** and **22b** adopt an “S” intermediate conformation that is converted to a “W” conformation like the open terpyridine when complexed to a second Cu^+^ cation. While the “S” state is obtained with one equivalent of copper(I), the full conversion to the “W” state is more difficult to achieve and needs up to 8 equivalents. This is due to the reduced basicity of the second pyrimidine’s nitrogen when the first is already coordinated. The py-pym-py closed-by-default “U”-shaped systems form host–guest complexes with electron-poor guests such as TNF. These tweezers are thus a three-state system controlled by copper(I) with allosterically regulated guest binding.

**Figure 14:**
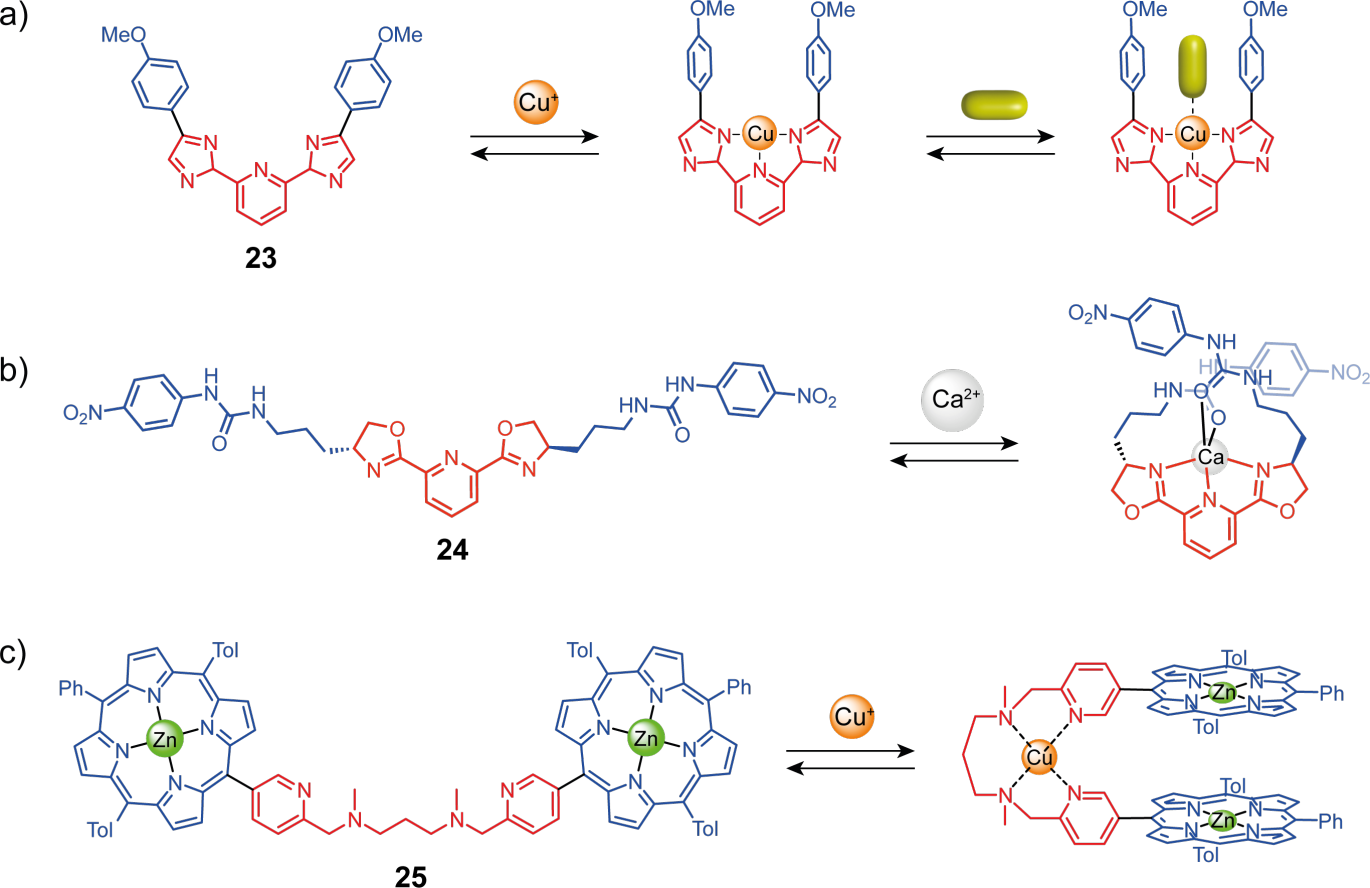
Coordination-responsive molecular tweezers based on nitrogen-containing ligands.

Other groups developed coordination-switchable molecular tweezers with several ligands based on nitrogen coordination sites. Plante and Glass reported tweezers **23** using a bisimidazole-pyridine unit with anisole arms (Figure 14a) [55]. This system can coordinate copper(II) in a square planar conformation giving an almost parallel arrangement of the aryl arms. This creates a cavity able to complex small coordinating guests that interact with the copper as ligands and also with the arms through π–π stacking. This system is selective towards flat aromatic guests and towards electron-rich coordinating guests. Detection by fluorescence was implemented using competition experiments with dimethylaminostyrylpyridine (DMASP). Intercalated DMASP is not emissive, but its displacement by a guest releases its free fluorescent form, which can be detected. The group of Nabeshima reported closely related tweezers based on a 2,6-bis(oxazolinyl)pyridine (Pybox) ligand with two 4-nitrophenylurea substituents as anion receptor **24** (Figure 14b) [56]. This ligand presents a “W” to “U”-shaped conformational change upon metal coordination similar to terpyridine but displays additional chirality that enables monitoring by circular dichroism. Upon the addition of Ca(II), a large increase in the binding affinity for halide ions was observed due to the folding of the receptor in a helicoidal form that enabled cooperative interaction with both urea moieties.

More flexible coordination responsive units composed of two pyridine units linked with a propyldiamine spacer (tweezers **25**, see Figure 14c) have been reported by Fuzukumi and co-workers [57]. This unit has four coordination sites and can bind copper in a square planar geometry. The authors functionalized this spacer with two zinc–porphyrin arms. In the uncoordinated state, the conformation is not fixed so the porphyrins do not interact but when the tweezers are closed the porphyrins are facing each other and can interact. The closed and open states exhibit different electrochemical and photochemical properties. The porphyrin interaction in the closed state splits the second oxidation of the porphyrins from a two-electron process to two single-electron processes. This is due to the electrostatic repulsion between the two positively charged complexes that shift the second oxidation potential. The absorption bands of the closed state are redshifted with respect to the open state, demonstrating the porphyrin–porphyrin interactions. Along with Lehn’s and Vives’ work [35,46], this example opens the way for new electroactive systems with a cation-controlled electrochemical behavior.

**Figure 15:**
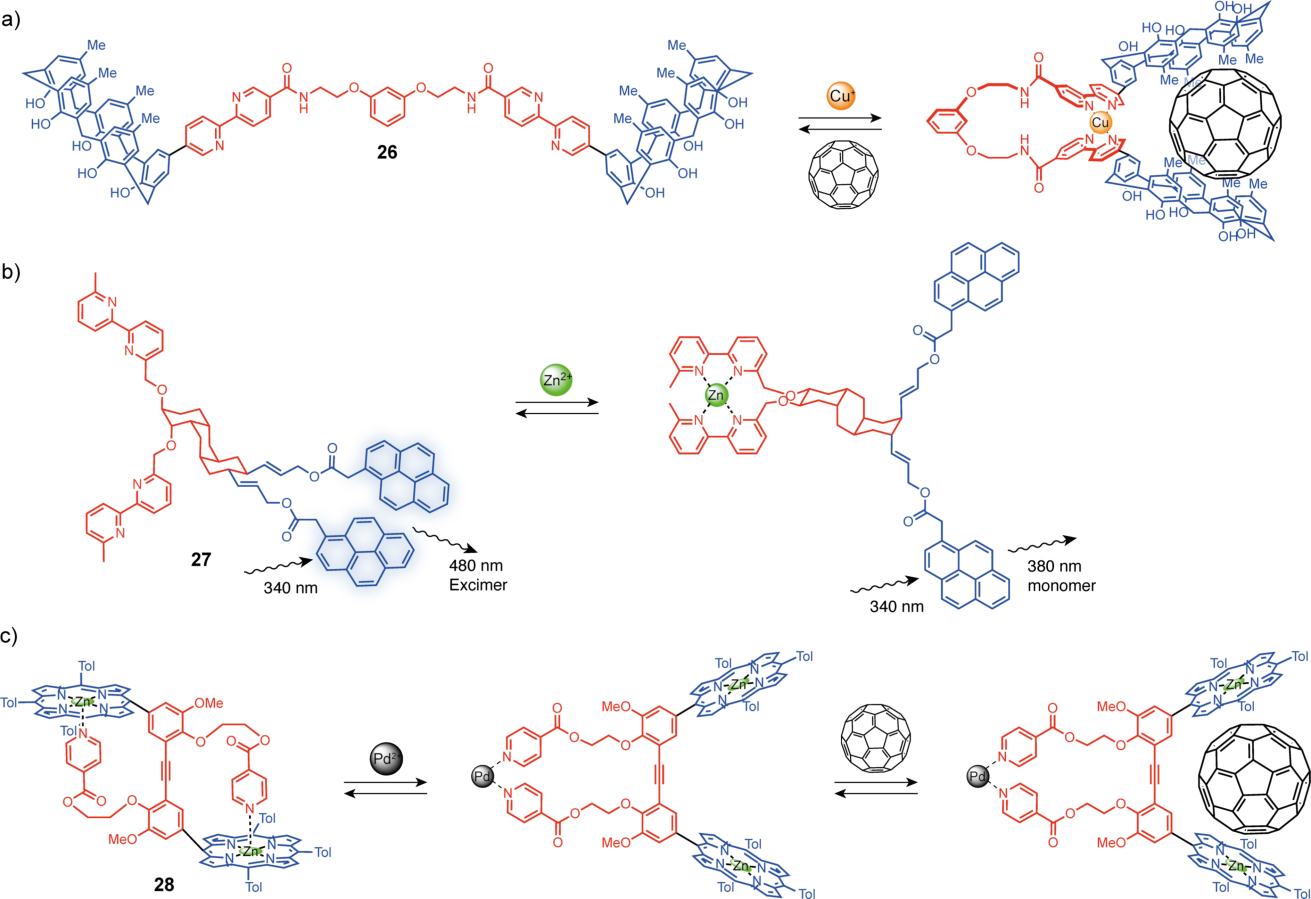
Molecular tweezers exploiting the remote bipyridine or pyridine binding to trigger the conformational change.

The common bidentate 2,2’-bipyridine ligand [58] has also been used as a switching unit for molecular tweezers. Like the terpyridine unit, substitution in 4 and 4’ or 6 and 6’ gives access to open or closed-by-default systems. An early example of allosterically regulated systems was reported by Fukazawa and co-workers. Their system **26** is composed of two bipyridine-calixarene units linked by a flexible spacer (Figure 15a) [59]. In the non-complexed state, the system presents no cavity. But, when copper(I) is introduced, a complex is formed with the two bipyridine units bringing the two calixarenes close to each other and creating a cavity suitable for C_60_ complexation. Bipyridine was also used in fluorescence signal transduction systems to induce a conformational change by coordination stimuli (Figure 15b) [60]. The tweezers-like system **27** is built around a tetrasubstituted *cis*-*anti*-*cis*-perhydroanthracene core functionalized at opposite ends with two bis-2,2’-bipyridyl ligands and bis-pyrene fluorescent units. The stable conformation of perhydroanthracene is with the two pyrene units in the equatorial position. Their close spatial proximity leads to the observation of an excimer emission. Upon Zn^2+^ coordination, the perhydroanthracene core undergoes a conformational change resulting in an axial position of the pyrene groups that are now far away and present monomeric emission properties. It should be noted that in both systems, the conformational change from s-*trans* to s-*cis* of the bipyridine unit is not directly responsible for the closing of the tweezers.

In a similar way but with monodentate pyridine moieties, Yamaguchi and co-workers described the bis zinc–porphyrin system **28** [61]. By default, the 1,1′-(1,2-ethynediyl)bis[3-methoxybenzene] spacer adopts a *trans*-conformation due to the pyridine units that intramolecularly complex the zinc in the apical position of the porphyrins (Figure 15c). Upon the addition of Pd(II), the two pyridine moieties prefer to coordinate the palladium which leads to a 180° rotation motion and confers a *cis*-conformation to the system. This motion positions the porphyrin units face-to-face and allows fullerene complexation in the cavity, thus enabling an allosteric regulation of the complexation properties of the system by a Pd(II) stimulus.

**Figure 16:**
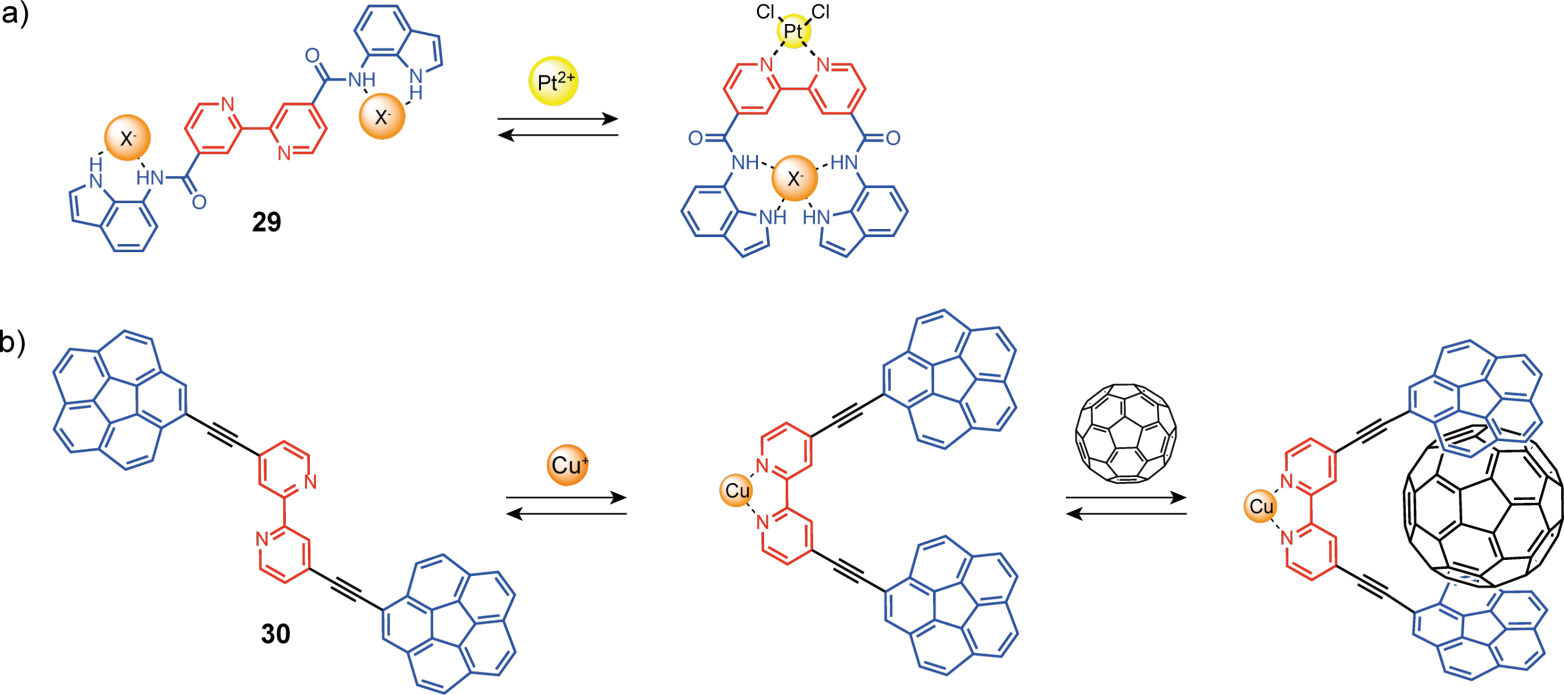
Bipyridine-based molecular tweezers exploiting the direct s-*trans* to s-*cis*-switching for a) anion binding or b) fullerene recognition.

An example of direct utilization of the rotation around the single bond of the bipyridine unit was reported by Caltagirone and co-workers in tweezers **29** (Figure 16a). They developed tweezers with carboxamidoindole units connected in 4 and 4’ positions to a 2,2’-bipyridine unit for switchable anion recognition [62]. The uncomplexed open conformation displays a very low affinity for anions such as chloride, acetate, or diphosphate (log K < 2). The coordination of PtCl_2_ preorganizes the tweezers in a closed conformation with the two hydrogen-bonding-recognition sites in proximity. A significant increase in the binding affinity toward all anions and in particular for dihydrogen phosphate (log K = 3.5) was obtained. More recently, Álvarez and co-workers reported bipyridine-based molecular tweezers **30** (Figure 16b) with corannulene recognition units on positions 4 and 4’ (open-by-default) for fullerene complexation [63]. The bipyridine can be switched from s-*trans* to s-*cis*-conformation by the addition of copper(II) and one equivalent of 1,2-bis(diphenylphosphino)ethane (dppe) ligand forming a square planar complex. This triggers the closing motion of the tweezers by rotation of one pyridine unit. The tweezers can be reversibly reopened by the addition of a second dppe equivalent that forms a more stable Cu(dppe)_2_ complex. The authors could successfully induce C_60_ and C_70_ complexation, with selectivity for C_70_ (log K_C60_ = 3.3 and log K_C70_ = 4.7).

In a follow-up of this work, they reported a similar redox-switchable biscorannulene tweezers system with a bis(arylthiol) switching unit that can be locked in the *cis*-conformation by forming a disulfide S–S bridge upon oxidation. As expected, the preorganized rigid form presents stronger binding affinities to fullerenes than the freely rotating dithiol form [64].

Recently, similar bipyridine-based tweezers with Zn–porphyrin functional units for fullerene binding were reported, using different stimuli for switching [65]. The Zn-closed tweezers were opened by adding H_2_PO_4_^−^ to competitively complex the Zn^2+^ ions. Then, Ca^2+^ was added to precipitate the hydrogen phosphate adduct and release the Zn^2+^ ions, closing the tweezers again.

**Figure 17:**
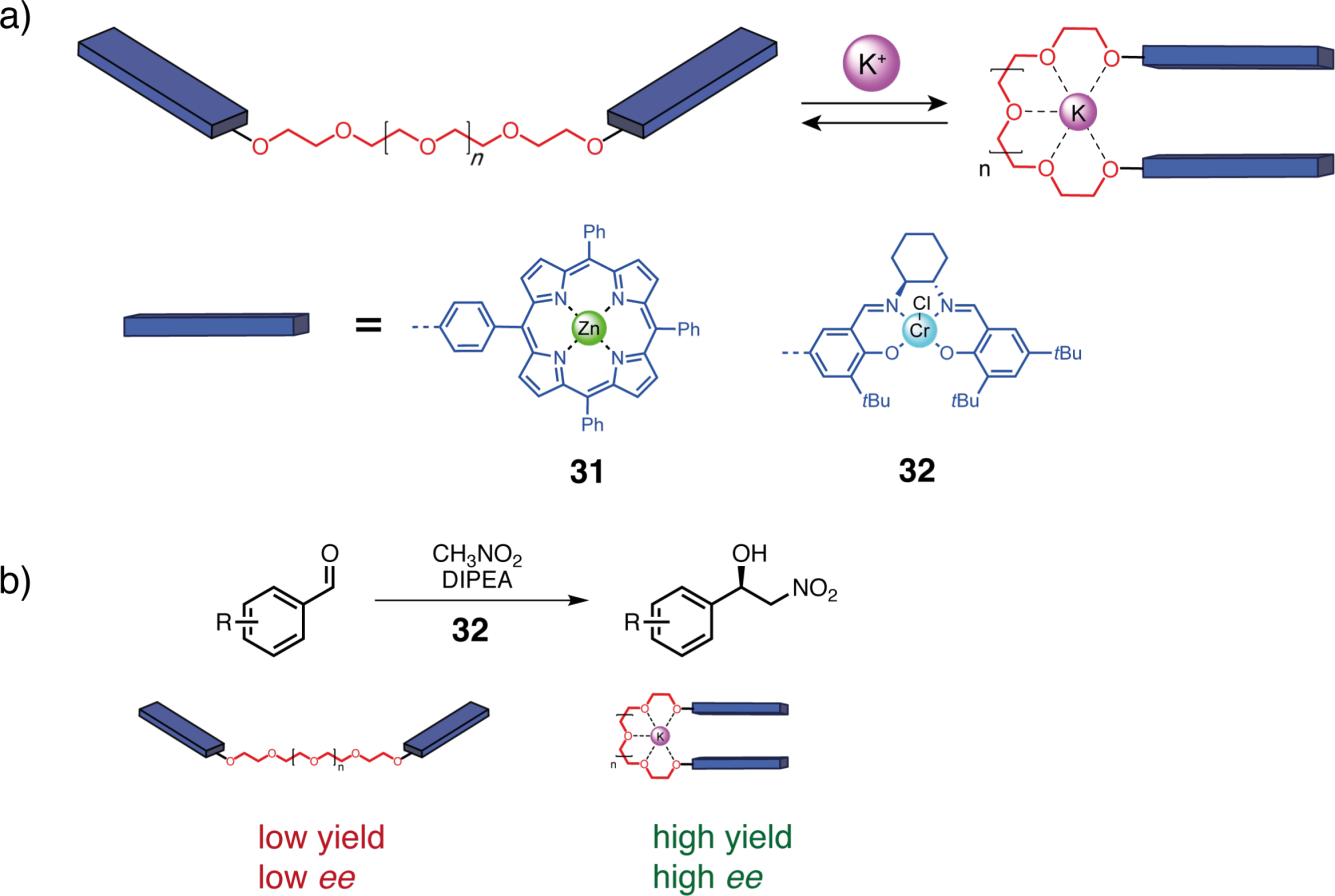
a) Podand-based molecular tweezers [66–67]. b) Application of tweezers **32** for the catalytic allosteric regulation of the Henry reaction between benzaldehyde derivatives and nitromethane.

Another class of a coordination-responsive spacer using oxygen coordination sites was developed concomitantly to the nitrogen-based systems. In 1999, Boschi and co-workers reported the podand-based switchable tweezers **31** (Figure 17) [66]. This kind of spacer is flexible due to the ethylene glycol chain but can complex alkali cations like crown ethers. Upon complexation, the podand unit adopts a cyclic conformation wrapping around the cation and brings the arms close to each other. The selectivity of the cation binding can be modulated by changing the chain length similarly to a crown ether (Figure 17). The cation can be removed by the addition of a better ligand such as a crown ether. The authors reported switchable tweezers bearing metalloporphyrin arms that can be switched by the addition of K^+^ and Na^+^ cations. The cation brings the porphyrins together and causes some dynamic fluorescence quenching from the iodine counter anion. The authors attributed the more efficient quenching using KI instead of NaI to the better size affinity of the podand with potassium.

Even though this system seemed promising, podand-based molecular tweezers have not attracted much interest for switchable molecular tweezers design until the work of Fan and co-workers in 2014 [67]. They reported tweezers **32** (Figure 17) incorporating chiral Cr(III)–salen arms for the allosteric regulation of a Henry reaction between benzaldehyde derivatives and nitromethane. In the closed form induced by K^+^ binding, high enantiomeric excesses around 90% and yields (<70%) were obtained due to the cooperative catalytic effect of the two Cr(III) units. Control experiments without K^+^, or in the presence of competitive crown ether presented a reduced conversion rate and enantioselectivity, proving the conformation change in the reactivity of the system.

### Anion responsive tweezers

**Figure 18:**
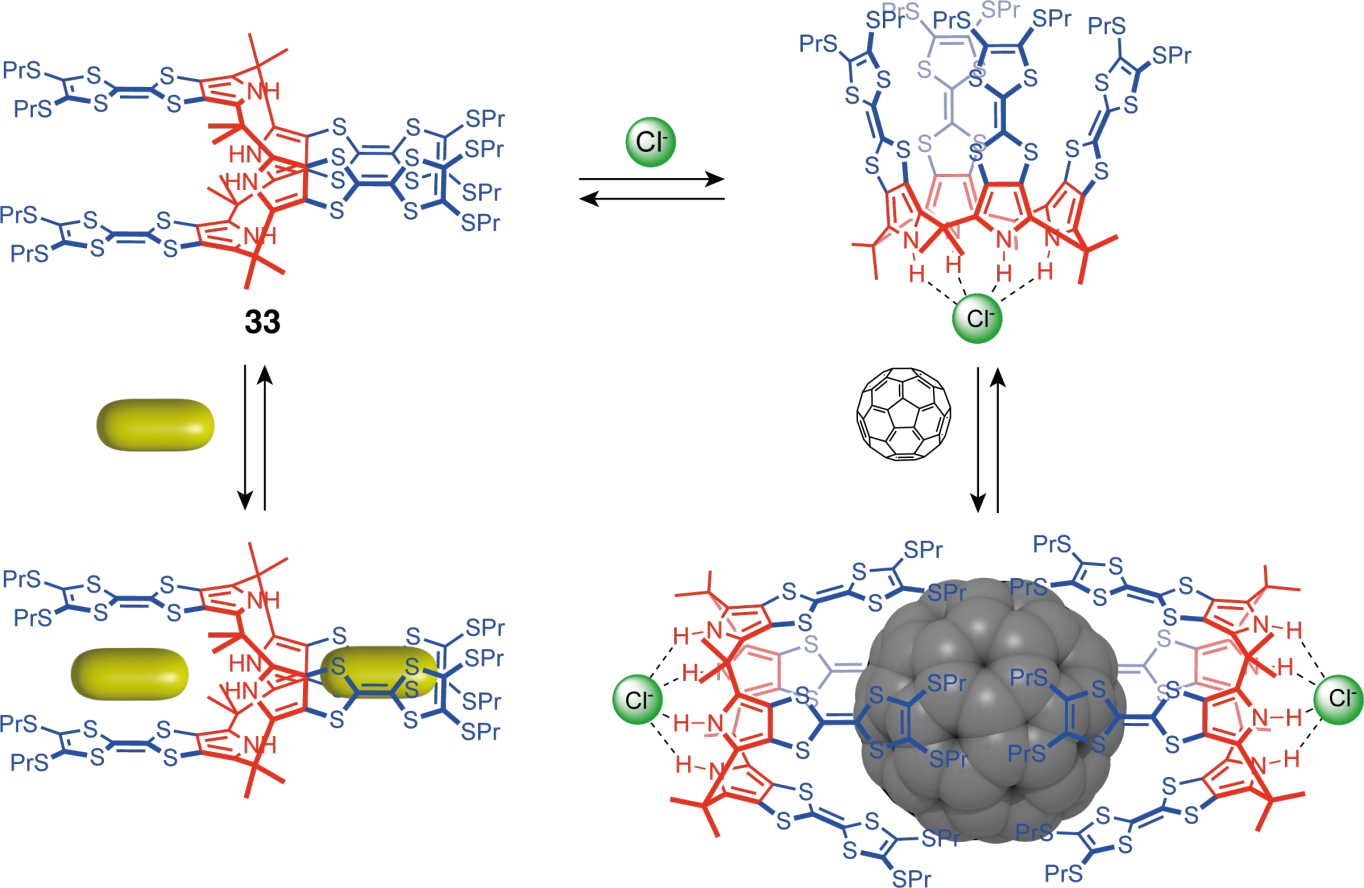
Anion-triggered molecular tweezers based on calix[4]pyrrole.

The main types of anion-responsive switchable tweezers are based on an H-bonding motif that can establish recognition interactions with the anionic species. One of the first examples was developed by Sessler, Jeppesen, and co-workers with a calix[4]pyrrole switching unit [68]. Calix[4]pyrrole tweezers **33** are functionalized by four tetrathiafulvalene (TTF) arms, adopting a 1,3-alternate conformation by default with two cavities for binding electron-poor guests between the TTFs (Figure 18). The authors demonstrated complexation with 1,3,5-trinitrobenzene and other electron-poor guests such as TCNQ. The N–H protons of the pyrrole subunits can form H-bonds with anions, causing the tweezers to switch to a cone conformation when Cl^−^ is introduced. This alters the cavities, allowing for the release of guests bound in the alternate conformation. Such a system offers new approaches towards molecular electronics by generating multistate systems with electronically differentiated states using the tweezers' switching and modulation of host–guest complexes' electronic properties [69–70]. Sessler also explored the host–guest properties of the cone conformation towards the complexation of fullerenes. Indeed, the large cavity of the cone-conformation calix[4]pyrrole and the electron-donating nature of TTFs allow the complexation of electron-poor C_60_ guests [71]. A 2:1 complexation of C_60_ by TTF-functionalized calix[4]pyrrole **33** was observed for the Cl^−^ bound cone conformation with each calix[4]pyrrole entrapping C_60_ on one side. The complex was easily detected by a large charge transfer absorption band (λ_max_ = 725 nm) and a noticeable emission change (brown to green solution). This complexation happened only in the presence of Cl^−^ and not with the tweezers in alternate conformation, demonstrating the allosteric regulation of the fullerene complexation. Other halides (Br^−^ and F^−^) also allowed C_60_ and C_70_ complexation while tetrabutylammonium cations inhibited the fullerenes' complexation by competing for the cavity of the calix [72]. These calixpyrrole-based tweezers provide a good example of ON/OFF molecular sensors for neutral guests with an anion-binding stimulus.

This system could further be tuned and adapted by increasing the macrocycle size or by modifying the TTF groups. Indeed, the authors later reported the allosterically regulated complexation of Li^+^ encapsulated C_60_ (Li^+^@C_60_) in TTF-calix[4]pyrroles and benzoTTF-calix[4]pyrroles [73]. The electrochemical properties of the host–guest system are modulated by a thermally induced electron transfer (ET) that generates the charge separation state [PrS-TTF-C4P^•+^/Li^+^@C_60_^•−^]. This behavior was first reported with Cl^−^ as an allosteric regulator but was then described with a porphyrin carboxylate anion as the effector. This allowed the generation of photoinduced charge-separated states with extended lifetimes in a supramolecular triad [74].

**Figure 19:**
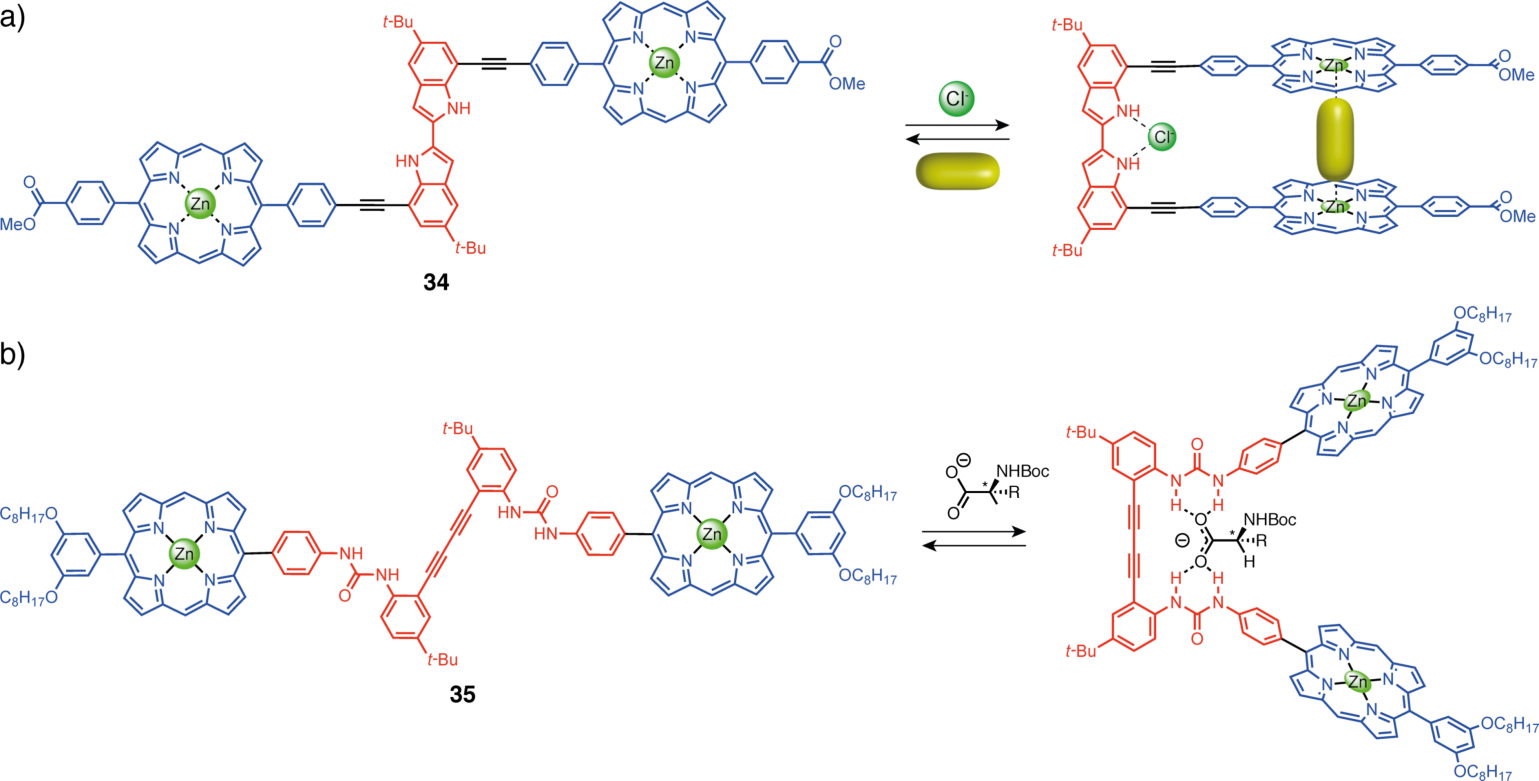
Anion-triggered molecular tweezers.

Another switching unit controlled by an anion-binding stimulus is bis-indole, reported by Jang and co-workers (Figure 19a) [75]. In the neutral state, the tweezers **34** adopt an *anti*-conformation because of steric hindrance between the N–H and the *tert*-butyl groups. Again, the N–H proton of the indole can bind to anions through hydrogen bonds and causes the system to adopt a *cis*-conformation, closing the tweezers. This closed form is able to bind diamine guests between the two porphyrins in the apical position for each zinc center. However, it has been observed that the closing can also be induced directly by diamine guests such as DABCO. This system exhibits an allosteric response with positive cooperativity as the binding constant is higher for the second complexation than the first. The authors later reported a similar system with triazole linkages between the porphyrin and the indoles that act as binding sites for the metal (square planar complex) [76]. These tweezers present a fluorescence quenching upon copper(I) addition because of a photoinduced energy transfer between porphyrins and the copper center. The fluorescence can be recovered by the addition of a bidentate pyrophosphate guest ligand, which disturbs the geometry of the system (octahedral complex) and thus lowers the energy transfer.

Jang and co-workers also developed tweezers **35** with a semi-rigid 1,4-diphenyl-1,3-butadiyne spacer containing two urea units linked to the aromatic rings and Zn–porphyrin functional units (Figure 19b) [77]. The arms have a free rotation around the rigid diethylene backbone and thus the system is natively in equilibrium between a *cis* and a *trans*-conformation. By the addition of an anion, the tweezers are locked in a closed form thanks to the H-bond formed with both urea moieties. The authors reported the closing of the tweezers using chiral carboxylates associated with circular dichroism (CD) response. In the presence of an achiral diamine (1,12-diaminododecane or DAD) that binds to both Zn–porphyrins and sets them further apart, a higher CD signal intensity is obtained making it a sensitive probe for determining the absolute stereochemistry of chiral carboxylates directly, without the need for derivatization. More generally, this type of switching unit can also be used with achiral anions to induce the closing of the tweezers and give access to the cavity between the porphyrins.

**Figure 20:**
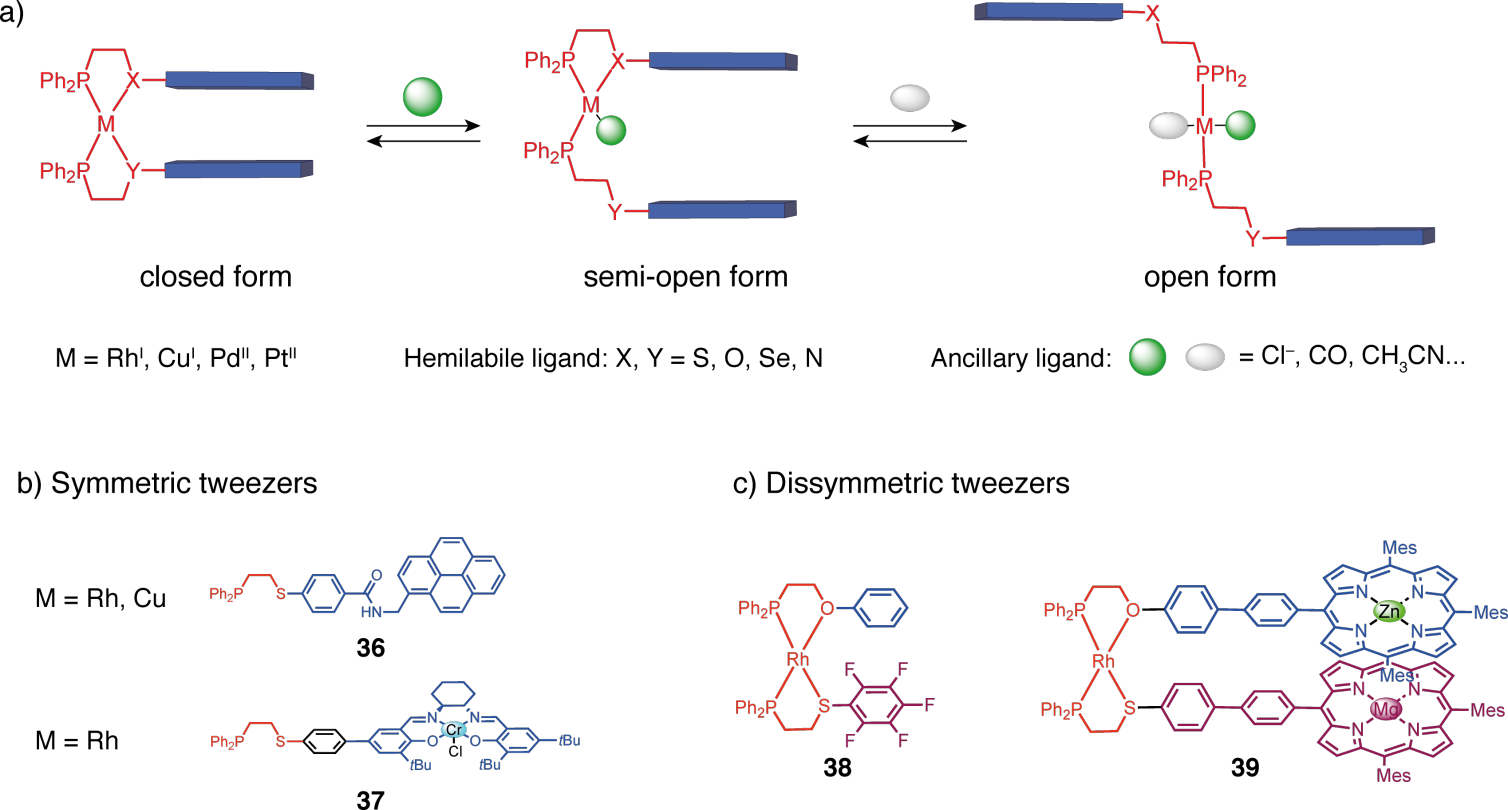
a) Principle of the weak link approach (WLA) developed by Mirkin and its application to b) symmetric and c) dissymmetric molecular tweezers.

In a quite different strategy from what we have described before, Mirkin and co-workers have introduced supramolecular switchable systems using metal complexes as switching units (Figure 20). This concept named “weak link approach” (WLA) [78] uses square planar d^8^-transition metal complexes with two hemilabile bidentate ligands composed of a strong binding site (phosphorus) and a weaker one (generally sulfur, oxygen, selenium, or nitrogen) that is labile. By default, the complex is in a closed form with the functional units on each ligand facing each other due to the square planar geometry of the complex. By adding a first ancillary ligand (carbon monoxide, chloride,…) that strongly binds to the metal center, one weak binding site is decoordinated and one arm is set loose, leading to the “semi-open” conformation. A second ancillary ligand needs to be added to decoordinate the labile binding site on the second ligand and fully open the system.

**Figure 21:**
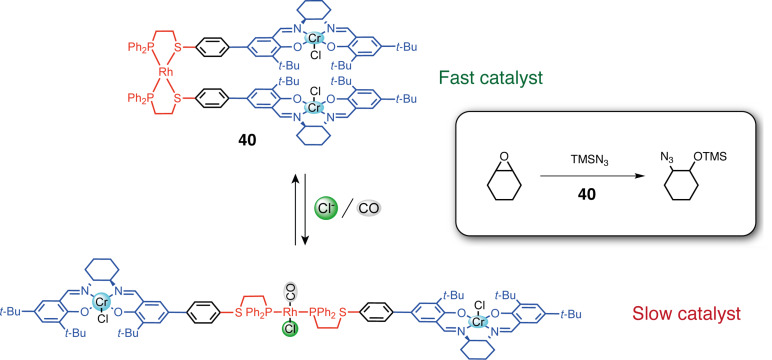
Molecular tweezers as allosteric catalyst in asymmetric epoxide opening [80].

Symmetrical tweezers can be obtained by using two identical hemilabile ligands. For example, tweezers **36** bearing pyrene fluorescent groups and using either rhodium(I) or copper(I) complexes as switching units were reported [79]. Cu(I), which avoids the luminescence quenching effect of Rh(I), requires pyridine as an ancillary ligand to complex the metal center and open the tweezers. The luminescence properties of the system could then be regulated by closing/opening but also by the addition of Cl^−^ that could form hydrogen bonding with the amide function of the spacer between the switching and the functional units. The WLA has also been applied to obtain switchable molecular tweezers for allosteric catalytic regulation (Figure 21). Mirkin and co-workers reported the symmetric tweezers **40** based on a Rh(I) complex with a phosphine and a labile thioether site and Cr(III)–salen arms as catalytic sites for the asymmetric ring opening of cyclohexene oxide by TMSN_3_ [80]. The closed tweezers showed higher activity and enantioselectivity due to the preorganization of the Cr(III) catalytic centers enabling bimetallic catalysis compared to the more flexible arrangement of the salen in the open form. The tweezers could be opened by breaking the thioether–rhodium bond with a combination of Cl^−^ and CO ligands. The reversibility of the opening/closing process was achieved in situ by the addition and removal of CO yielding one of the first examples of allosteric regulation in artificial systems.

**Figure 22:**
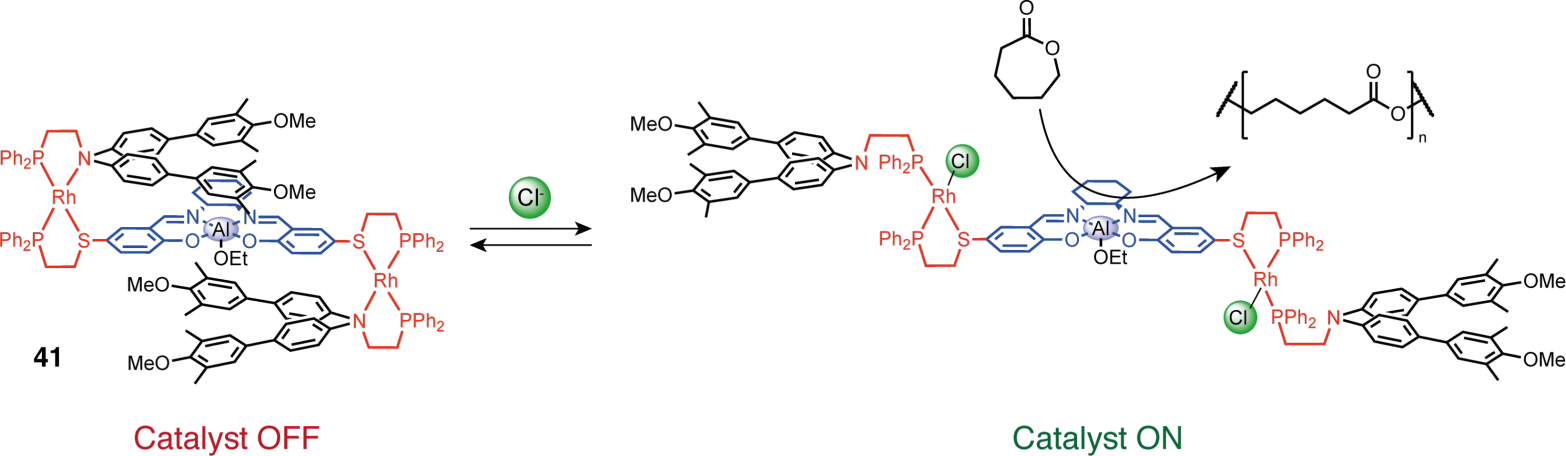
Allosteric regulation of catalytic activity in ring-opening polymerization with double tweezers **41**.

Dissymmetric molecular tweezers are also accessible with high selectivity using two different hemilabile ligands and a halide-induced ligand rearrangement (HRI) [81]. For example, when two symmetric Rh(I) complexes, one with P,O and one with P,S ligands, are subjected to the addition of halide anions, a rearrangement is observed by ligand exchange from the symmetric [Rh(P,O)_2_], [Rh(P,S)_2_] complexes to the dissymmetric [Rh(P,O)(P,S)] complex. This rearrangement allows access to tweezers with two different arms with two types of weak sites of different reactivities. This HRI reaction was first used to access simple dissymmetric rhodium(I) tweezers such as **38** with different aromatic arms [82], **39** with Zn/Mg metalloporphyrin arms [83] or larger assemblies [78]. In particular, remarkable double tweezers (or triple-decker catalysts) **41** have been developed (Figure 22). These tweezers consist of two Rh(I) complexes, wherein a catalytically active metal Al(III)–salen arm is shared on the phosphine thioether ligand side, and two separate aromatic arms are present on the phosphine ether/amine ligand side [84]. In the closed form, the system is catalytically inactive because the bulky aromatic arms stack with the salen complex and shield its access to the substrate. The addition of Cl^−^ or acetonitrile causes the protecting arms to move away from the salen complex thereby exposing the catalytic site. This triple-layer catalyst was applied in the control of a ring-opening polymerization reaction. The open state achieved full monomer conversion, while the closed state exhibited minimal activity (7% conversion after 100 hours).

Another class of dissymmetric tweezers was later developed based on square planar Pt(II)-based hinges [85–86]. The heteroleptic Pt(II) complexes were directly obtained by coordination of two different phosphine thioether ligands with either aliphatic or aryl substituents. The hemi-opening of such Pt(II) dissymmetric tweezers could be controlled as aryl thioethers are more labile than alkyl thioethers. The addition of Cl^−^ thus first breaks the Pt–SAr bond. Platinum-based dissymmetric tweezers were also synthetized using NHC thioether ligands that are more stable than the phosphine thioether ligands. This strategy led to elaborated dissymmetric systems that have been used as switchable chromophores [87] and in counterion-controlled phase-transfer processes [88].

To summarize, coordination-responsive molecular tweezers have been developed using a variety of switching units with a predominance of cation-responsive systems using nitrogen coordination sites. However, other types of systems with new approaches have appeared and now offer new tools for the development of new stimuli-responsive supramolecular systems. They have proved their potential in diverse potential applications from allosterically regulated host–guest systems to catalysis or magnetic properties.

### Redox switchable molecular tweezers

**Figure 23:**
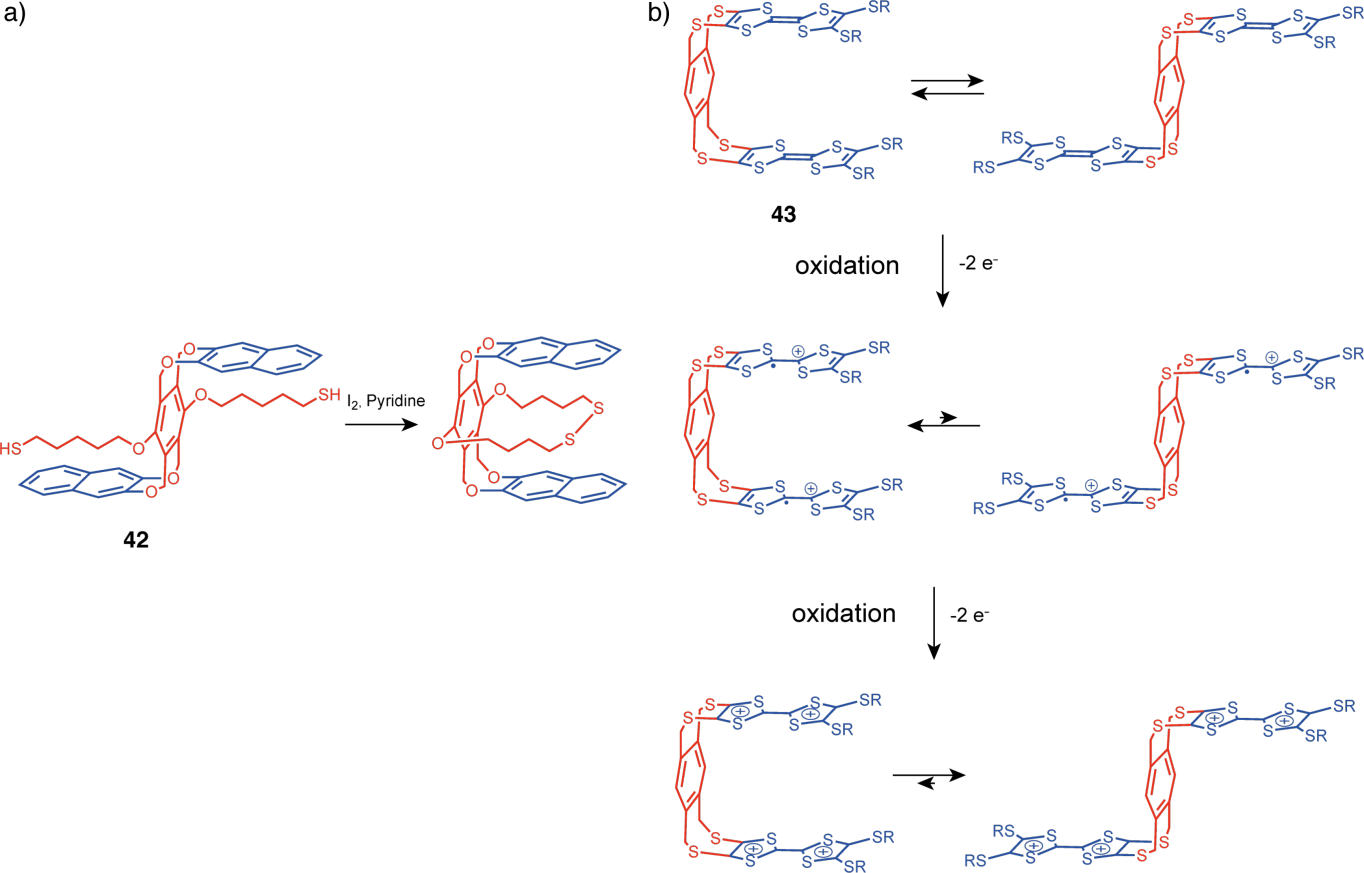
a) Conformational switching of **42** by intramolecular –S–S– bridge formation. b) Shift of conformational equilibrium of tweezers **43** by oxidation.

One of the cleanest stimuli for supramolecular switching is electron transfer. Besides photoswitching, the undeniable advantages of redox switching are the absence of chemical waste and the fact that no new atoms are introduced into the molecule, thus ensuring first-order kinetics of the process without complexity. One of the possible mechanisms of redox-induced switching involves an intramolecular bond formation that introduces non-covalent interactions between tweezers endpoints leading to a preferred conformation. It was achieved in tweezers **42** by the formation of an intramolecular disulfide bridge, that enables non-covalent bonds with aromatic moieties and results in a favored *syn*-conformation (Figure 23a) [89]. Yet, the most explored way is to use an electroactive moiety that can change its electronic or geometrical properties upon oxidation or reduction.

#### TTF and derivatives tweezers

One of the most widely used electroactive moieties is tetrathiafulvalene (TTF). Its electron-donor π-system can form non-covalent interactions with various electron-poor π-systems, and those interactions can be switched off by the TTF oxidation at low potentials, leading to electrostatic repulsion between the positively charged TTF and electron-deficient acceptor counterparts [90].

The conformation of such structures was studied by the Azov group using TTF tweezers **43** with flexible arms [91]. In the neutral state, conformational analysis performed by variable temperature NMR led to the conclusion that the open and closed conformations have similar energies and are in fast exchange at ambient temperature. This fluxionality resulted in a relatively low affinity for the electron-poor aromatic guest 2,4,7-trinitro-9-fluorenylidenemalonitrile (*K* = 16 M^−1^ in CDCl_3_). Upon single oxidation of both TTF, the voltammogram is characteristic of two TTF units in close proximity hinting at a favored closed conformation. However, in the fully oxidized state, the two TTF units are no longer in interaction and at a larger distance due to electrostatic repulsion between the doubly charged redox-active units. Thus, it can be concluded that, although the tweezers exhibit different conformations in neutral and oxidized states, at room temperature both states are in equilibrium and the potential barrier is lower than thermal energy at 298 K, which is a limitation for using this system as efficient redox-responsive tweezers (Figure 23b).

**Figure 24:**
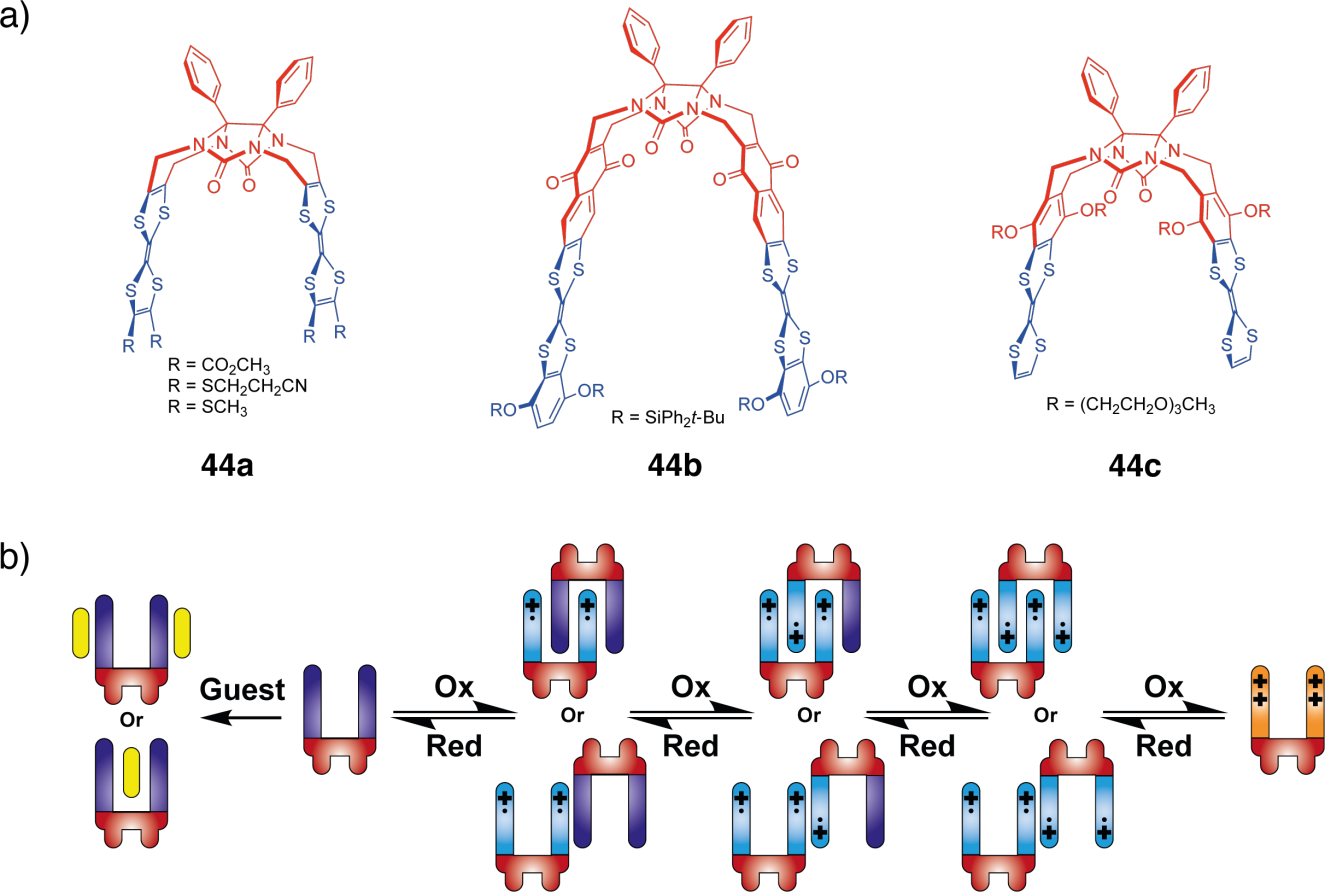
a) Redox-active glycoluril-TTF tweezers **44**. b) Mechanism of stepwise oxidation of said tweezers with formation of the dimers.

As observed in **43**, when oxidized to a radical cation state, TTF can form mixed-valence dimers that are held together by radical bonds [92]. However, these dimers are too unstable in solution for the free TTF to be detected. To stabilize the dimers in solution the groups of Chiu [92] and Hudhomme [93–94] designed molecular tweezers **44a**–**c** with a flexible central unit based on a glycoluril moiety (Figure 24a). These molecules can have geometrically multiple interactions with each other, thus further increasing the enthalpic contribution per molecule of mixed-valence and radical cation dimers and hence, improving their stability in solution. The oxidation of the designed molecular tweezers with TTF functional groups was studied and the authors observed that upon oxidation TTF tweezers form dimers, stabilized through a combination of mixed-valence and radical cation interactions, that are strong enough to be observed in solution (Figure 24b).

**Figure 25:**
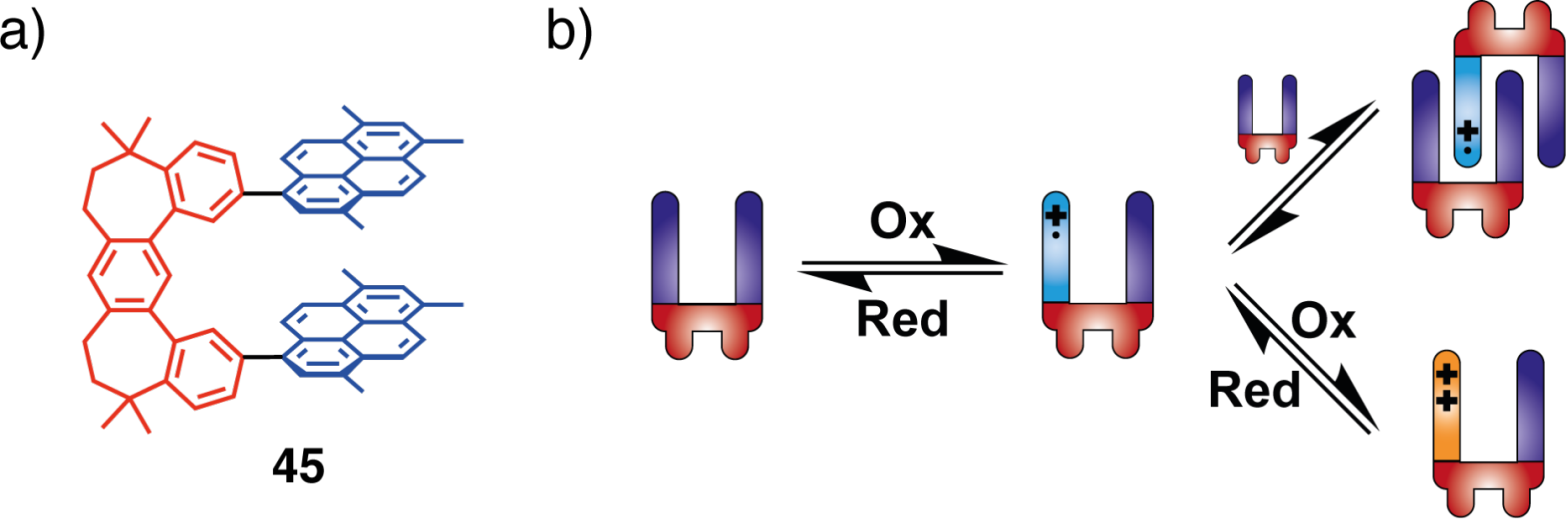
Mechanism of formation of the mixed-valence dimers of tweezers **45**.

Very similar behavior was observed by Rathore et al. for their tweezers with pyrene redox units **45** [95]. In addition to the observed formation of a cation radical and dication with electrochemical and chemical oxidation, the authors managed to form an interlocked mixed-valence dimer (Figure 25) upon the addition of neutral tweezers to its radical cation solution. Such dimer was detected by the NIR absorption band (>1400 nm and λ_max_ = 2620 nm).

**Figure 26:**
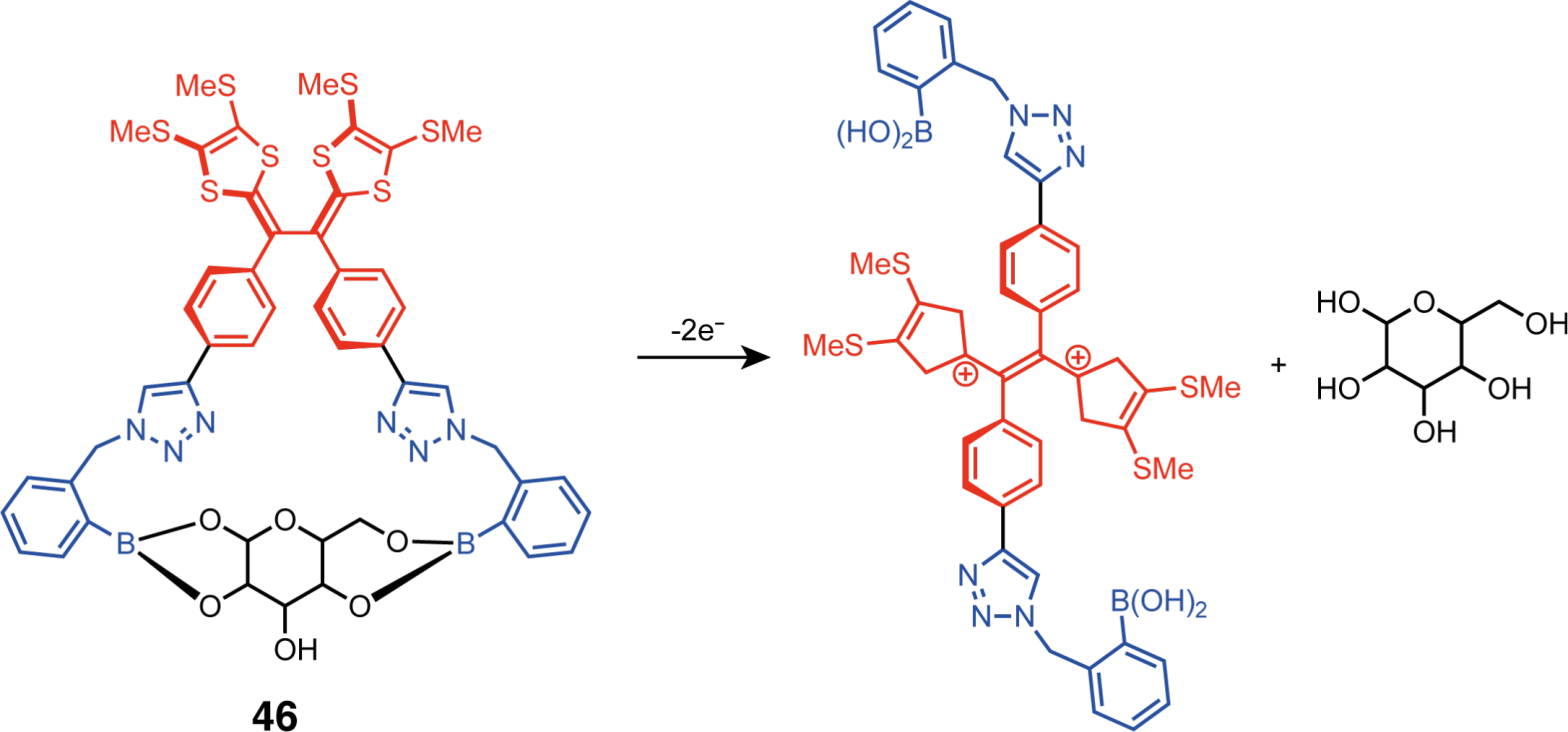
Mechanism of carbohydrate liberation upon redox-mediated conformation switching of **46**.

A significant limitation associated with the TTF moiety as a redox switching unit is its limited capacity for inducing geometry changes upon oxidation. TTF-based tweezers heavily depend on the π-stacking interactions of their arms, which are typically comprised of TTF derivatives. This leaves minimal opportunities for arm functionalization and necessitates the incorporation of a flexible central unit. In contrast to TTF, its vinylogue TTFV has the unique property to significantly change its shape upon oxidation. This allows to design of tweezers with the redox-active moiety as a rigid central unit. The group of Zhao has developed molecular tweezers with this rigid TTFV central switching unit which can change its conformation with a redox stimulus (Figure 26) [96]. The switching was monitored by electrochemical methods by comparing it to the behavior of TTFV described in the literature [97]. Also, the same group expanded the concept of functional TTFV tweezers as electrochemical sensors for selected carbohydrates [98]. Tweezers **46** have a specific electrochemical response to the presence of ribose and fructose, initially forming a 1:1 covalent complex, which has a slightly higher oxidation potential than the free tweezers due to the need to break covalent bonds during the conversion to the open conformation upon oxidation. As the saccharide concentration increases, the 2:1 complex becomes more prevalent, which is accompanied by the facilitation of the oxidation process, thus lowering its redox potential.

**Figure 27:**
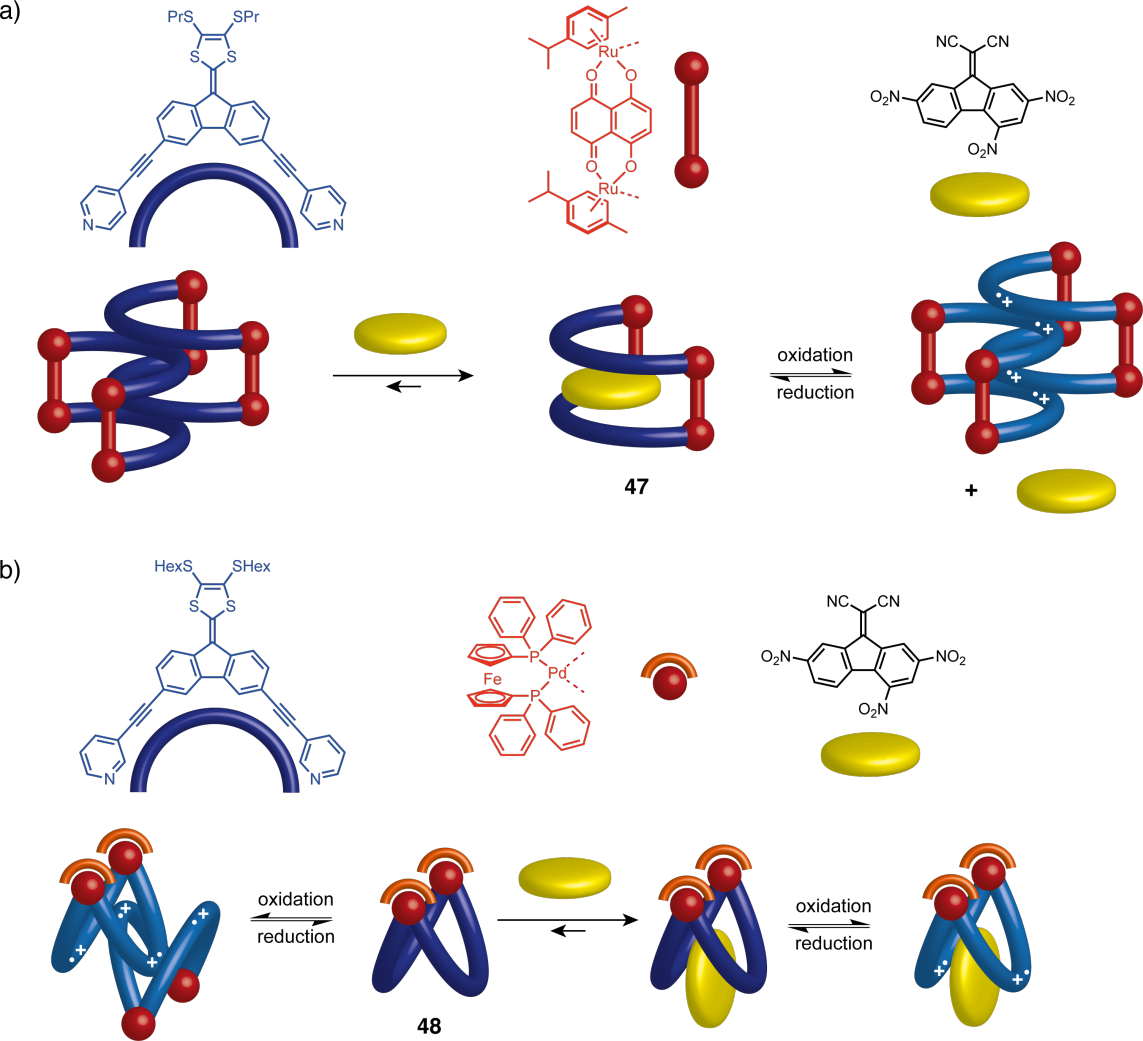
a) The encapsulation properties of **47** as well as the DCTNF release process from its host–guest complex with the concomitant formation of the oxidized dimer. b) The redox behavior and binding properties of **48**.

Besides covalent organic molecules, another category of tweezers-like structures can be self-assembled from planar, banana-shaped ligands and metal acceptors possessing the desired coordination geometry. These stimuli-responsive structures incorporate electron-rich 9-(1,3-dithiol-2-ylidene)fluorene (DTF) units and have recently been developed by Goeb et al. [99–101]. The overall geometry is determined by the choice of metal acceptor which can be a half-sandwich dinuclear ruthenium [99] or a *cis*-blocked palladium [100] complex. An intramolecular distance between the arms of approximately 8 Å is obtained to allow radical-assisted dimerization upon oxidation, a phenomenon previously described for TTF-based molecular tweezers. Electrochemical studies have demonstrated that **47** and **48** also possess the ability to encapsulate planar, electron-poor aromatic guest molecules (Figure 27). Furthermore, **47** can release these guest molecules upon oxidation of the tweezers (Figure 27a), presenting a concept of controlled guest release through self-assembled molecular tweezers.

#### Bispyridinium-based tweezers

**Figure 28:**
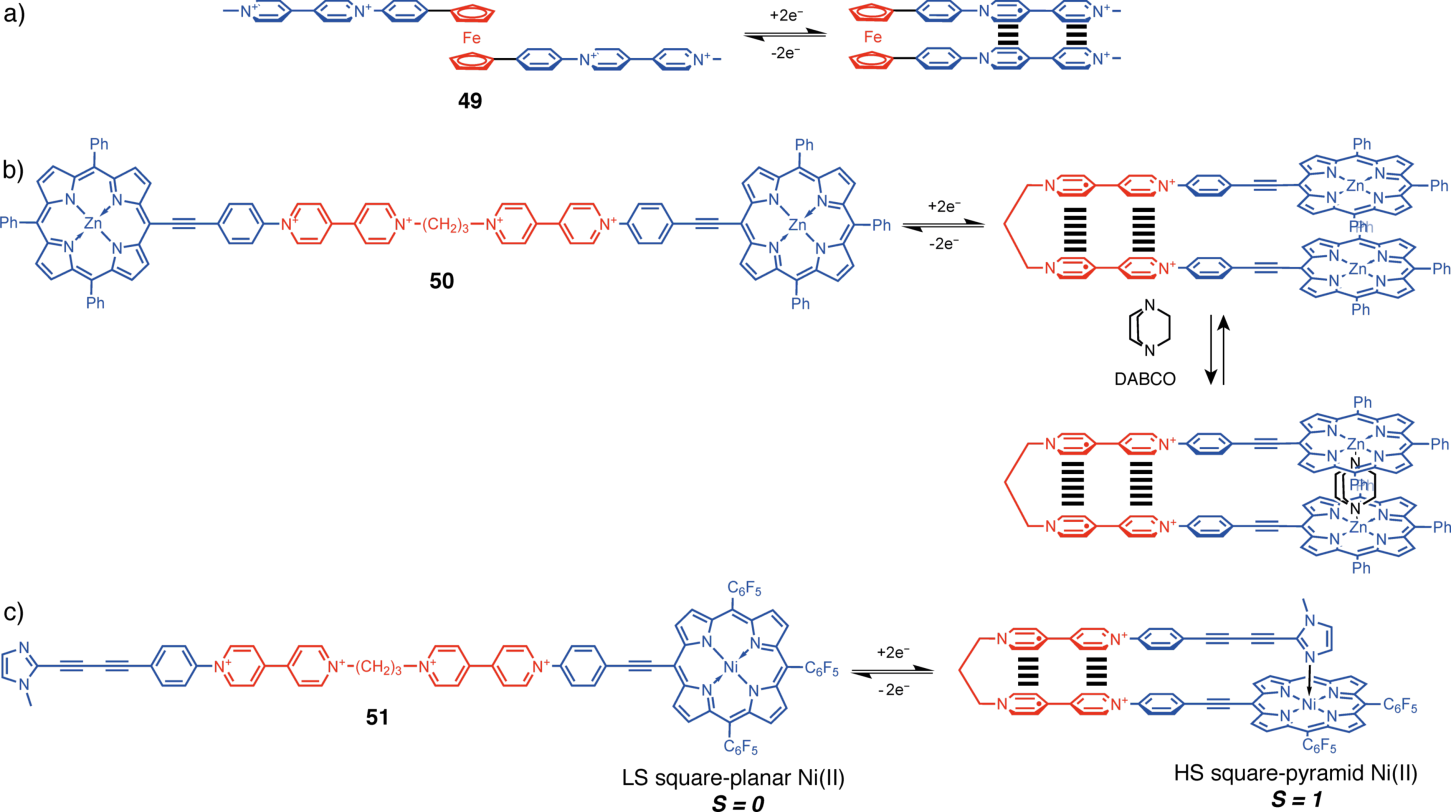
Redox-active bipyridinium-based tweezers. a) With a ferrocenyl hinge **49**, b) with a propyl hinge **50** enabling guest binding in the closed form, and c) dissymmetric tweezers **51** displaying spin-crossover response.

Another functional moiety, capable of self-stacking, controlled by redox stimulus, is the electron-acceptor bipyridinium moiety. This moiety has received huge research interest in supramolecular chemistry due to its redox-controlled stacking/repulsion cycle, very useful for molecular switches based on rotaxane and catenane architectures [102]. An example of its exploitation in redox-active molecular tweezers has been described by Bucher et al. [103]. A combination of bipyridinium functional units and a ferrocene central hinge was utilized for the development of flexible molecular tweezers **49** that are able to switch between their closed and open conformations with a redox stimulus (Figure 28a). In comparison to non-interacting analogs, the lower Δ*E*_p_ value of the first reduction process and the shift of the second viologen-centered reduction at a less negative potential value indicates that the two bipyridinium centers do not behave independently and form a π-dimer complex in the reduced state. The apparition of a π-dimer was also confirmed by UV–vis absorption, namely, by the apparition of a characteristic NIR band of π-dimers at 900 nm. Thus, in the tetracationic state the ferrocene tweezers adopt an open conformation due to the electrostatic repulsion between the bipyridinium units. Upon reduction, the corresponding radical anions start to interact with each other, leading to the turning of the ferrocene unit and occupying the closed conformation.

Another way to switch the conformation is with a flexible spacer. The tweezers **50**, with a flexible propylene spacer, bipyridinium functional arms, and porphyrin endpoints, were also developed by Bucher et al. (Figure 28b) [104]. The conformation change in this case can also be easily monitored by electrochemical methods. Upon reduction, UV–vis absorption, reveals the apparition of the characteristic NIR band of π-dimers at 900 nm and the characteristic exciton coupling between porphyrin chromophores which is evidenced by the hypsochromic and hypochromic shifts of porphyrin Soret band at 425 nm. In the closed form, the binding of a DABCO guest molecule between the two Zn–porphyrin arms of the tweezers was achieved. In the extension of this work, dissymmetric tweezers bearing Zn–porphyrin and imidazole-terminated arms were developed. An electron-triggered switching between a folded monomer in the reduced closed state and a coordination polymer in the open oxidized state was achieved [105].

Further development of this concept allowed Bucher et al. to develop redox-switchable coordination-induced spin-state crossover operating on the intramolecular self-locking principle [106]. The dissymmetric tweezers **51** comprise a Ni(II)–porphyrin bipyridinium subunit and an imidazole bipyridinium subunit, introduced on both arms, which are connected through a flexible propylene linker. Thus, upon reduction of the bipyridinium moieties, **51** switches its conformation to a closed one with the imidazole ligand coordinating to the nickel metal center, altering its coordination geometry from low spin square planar to high spin square pyramid (Figure 28c). The ability of the imidazole ligand to effectively coordinate in the apical position to the Ni(II) center was confirmed by the bathochromic shift of the Soret band proceeding through a well-defined isosbestic point at 436 nm. The spin-crossing was confirmed by paramagnetic NMR studies.

#### Redox-active porphyrin-based tweezers

**Figure 29:**
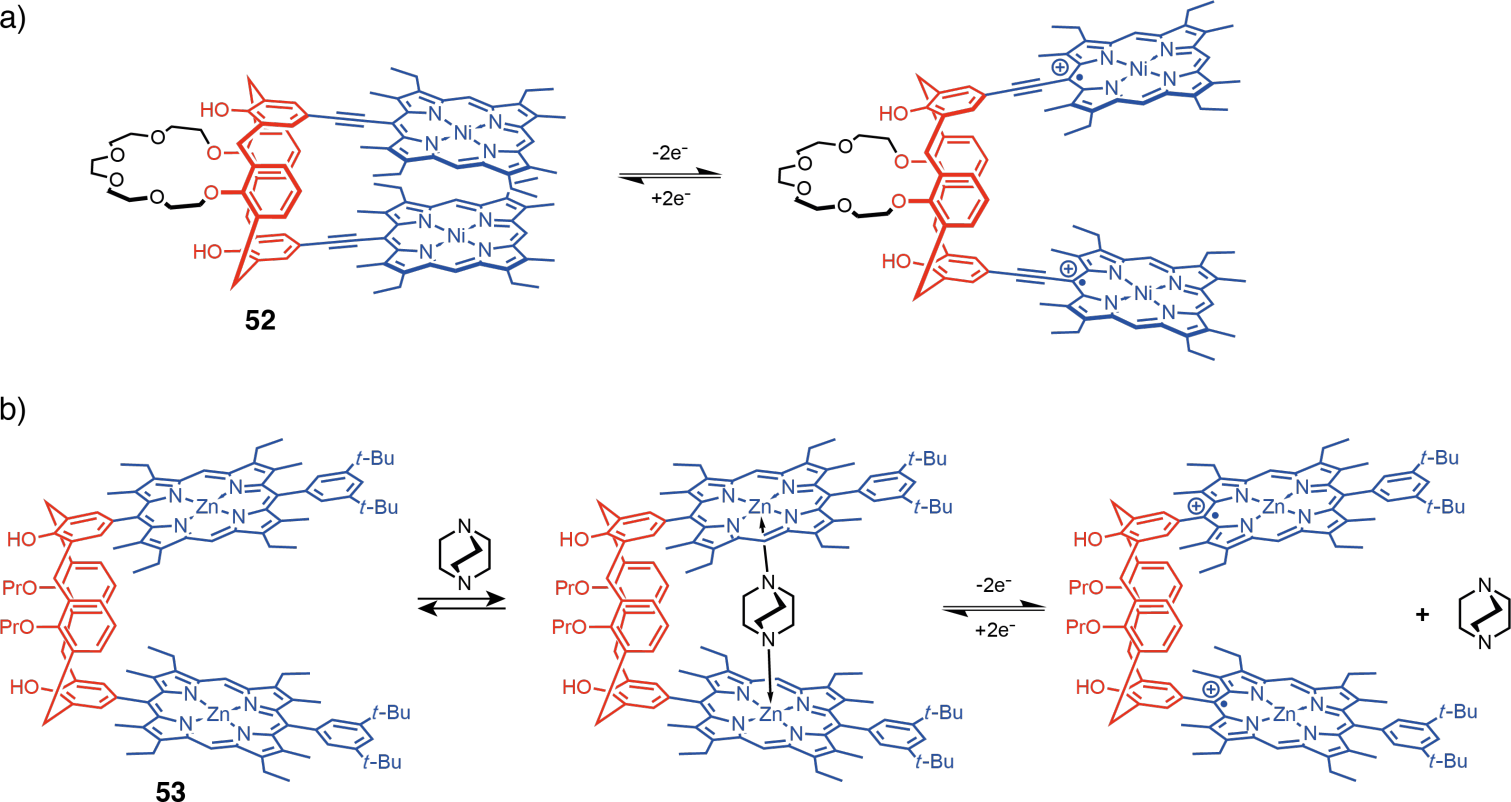
Redox-active calix[4]arene porphyrin molecular tweezers.

Porphyrin moieties can also be used as redox non-innocent units to change the shape and tune guest encapsulation properties of molecular tweezers. One-electron oxidation of a porphyrin moiety impacts the ability of the metal atom to form dative bonds with Lewis bases, which can be exploited to modify affinities to guest molecules. Weiss et al. reported calix[4]arene-based pacman Ni–porphyrins **52** (Figure 29a) where the distance between the two porphyrin arms was modulated by a redox stimulus [107]. In the neutral state, the calixarene enables a cofacial preorganization of two porphyrins that can stack and close the tweezers. Upon oxidation, the electrostatic repulsion between the two positively charged porphyrins opens the tweezers cavity. This system thus displays a redox-controlled handclapping-like motion. The analogous tweezers with Zn–porphyrin were then exploited to control the binding of bridging guests such as DABCO or pyrazine [108]. More recently Arimura et al. reported a related calix[4]arene-based tweezers **53** [109]. Here the intercalated DABCO guest could be released upon electrochemical oxidation due to the alteration of electronic properties of the metal center of the porphyrin (Figure 29b).

**Figure 30:**
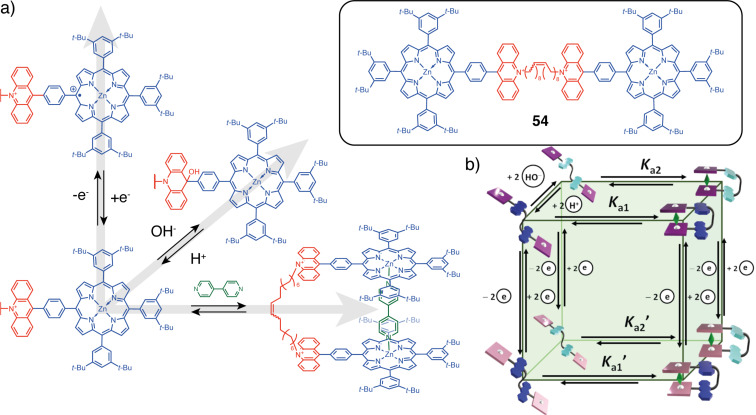
a) Mechanism of the three orthogonal stimuli. b) Cubic scheme showing the eight different states of **54** upon addition of 4,4′-bipy, nucleophiles, and electrons. Figure 30 was reproduced from [110] (© 2023 A. Edo-Osagie et al., published by American Chemical Society, distributed under the terms of the Creative Commons Attribution-NonCommercial-NoDerivs 4.0 International license, https://creativecommons.org/licenses/by-nc-nd/4.0/). This content is not subject to CC BY 4.0.

A similar strategy for controlling guest encapsulation was developed by de Rouville, Heitz et al. They used a flexible architecture to modulate the photochemical properties of porphyrin-based tweezers **54** (Figure 30a) and their affinity to bipyridine guests [110]. Specifically, **54** also incorporates the pH-switchable acridinium unit discussed previously, which introduces another orthogonal pH stimulus into the functional material. This results in a multistate system with eight distinct states with different photochemical properties and different affinities to the bipyridine guest molecule (Figure 30b).

#### Diquinone-based tweezers

**Figure 31:**
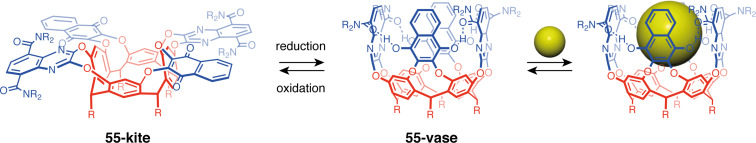
Redox-controlled molecular gripper based on a diquinone resorcin[4]arene.

Resorcin[4]arene are 3D macrocycles able to adopt two spatially well-defined conformations: an expanded kite and a contracted vase (Figure 31). This switching behavior was first observed in response to a temperature change [111]. The favorable solvation energy stabilizes the kite form at low temperature while the vase form becomes predominant at high temperature. The response to other stimuli such as pH, metal coordination, solvent, or guest binding has been later explored mainly by Diederich and co-workers [112–113]. Redox switching in the diquinone-based resorcin[4]arene cavitand **55** proved to be one of the most efficient stimuli [114]. In the quinone form, the vase form is strongly destabilized due to steric repulsion between the amide and the quinone moieties. Upon reduction of the quinone to hydroquinone, the vase conformation becomes stabilized by intramolecular hydrogen bonds between the hydroxy and the amide groups. The conformational change can thus be reversibly controlled by a redox stimulus. A large variety of hydrocarbon guests could be encapsulated in the closed vase conformation and subsequently released upon oxidation of the cavitand walls. This system illustrates how a redox-triggered mechanical motion on a receptor with extremely different molecular geometries can be harnessed to develop a molecular switch with clear ON/OFF binding properties.

### Photoresponsive molecular tweezers

Light is a choice stimulus due to its spatial and temporal resolution which has been thoroughly explored in photochromic-based molecular switches. Among photoresponsive moieties, only a few display large conformational changes between the two forms that are suitable for switchable molecular tweezers. They include azobenzenes, stilbenes, or hemi-indigos which will be presented in the following. Since photoresponsive molecular tweezers for molecular recognition have been recently reviewed by Wezenberg et al. [115], we will focus, in this section, on selected examples with an emphasis on other applications.

#### Azobenzene-based tweezers

Azobenzenes are a class of organic compounds characterized by the presence of an azo group that connects two benzene rings. The double bond between two nitrogen atoms can isomerize from *cis* to *trans* upon irradiation, leading to reversible structural and electronic changes. The extensive research that has already been conducted on their scaffold is a key advantage to their use [116–119]. Their fully reversible photoisomerization with visible light renders them valuable in various applications such as molecular switches and photopharmacological agents.

**Figure 32:**
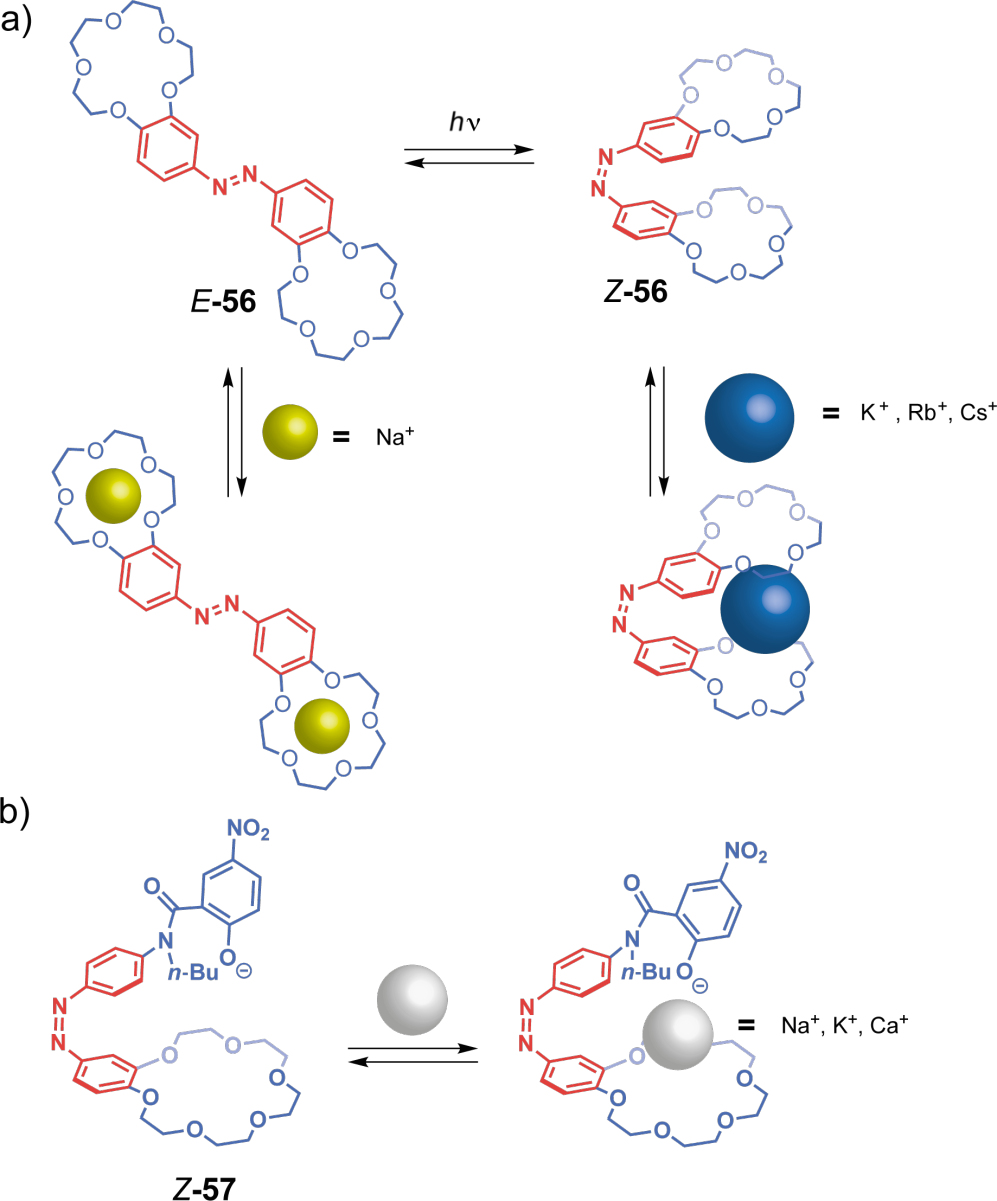
a) Shinkai's butterfly tweezers and their different host–guest properties depending on the isomer. b) Shinkai's dissymmetric tweezers.

In a seminal work, Shinkai et al. developed in the eighties bis-crown ether tweezers bridged with an azobenzene unit for the complexation of alkali metal ions [16,120]. His work on photoresponsive tweezers has since been famously known for their butterfly-like movements as the reversible switching between the *E-***56** and *Z*-**56** isomers (Figure 32a) upon irradiation closely reminds of butterfly motions. Since alkali ions usually form 1:1 or 1:2 complexes with 15-crown-5 ethers, depending on their ionic radii, the authors evaluated the ion extractability for each isomer of tweezers **56**. As expected, *E*-**56** presented a better affinity for sodium ions than its *Z* counterpart forming a 1:2 complex. However, the *cis*-form effectively recognized larger cations such as K^+^, Rb^+^, and Cs^+^ in a 1:1 assembly. Moreover, *cis-*to-*trans* thermal isomerization, which occurred fairly fast in the dark (approximately 10 min), was quelled thanks to the significant stability gain of the sandwich complex in the tweezers *cis-*form. These tweezers were exploited in a cation-transport experiment across an organic layer. The azobenzene moiety serves subsequently as an effector to control the release of these guests upon a phototrigger after transport.

In 1982, Shinkai attempted a dissymmetric version **57** of the previous system [121], with an 18-crown-6 functional unit on one part and a phenoxide on the other (Figure 32b), to achieve ionophoric properties. Indeed, the phenoxide acts as an anion cap that can be switched on or off upon photoisomerization of the azobenzene moiety. These tweezers also show more efficient ion transport since the formed complexes’ stability is moderate, thus allowing guest release more easily.

More recently, anion-controlled isomerization of an azobenzene receptor was reported by Dąbrowa and Jurczak with phenylurea recognition units [122]. The *Z*-isomer binds in a 1:1 stoichiometry to anions such as F^−^, AcO^−^, and H_2_PO_4_^−^. However, the increase in anion concentration enhances significantly the thermal *Z-*to*-E* isomerization rate and induces the formation of an open 1:2 complex. The latter displays a higher binding affinity for the substrates, especially for the acetate ions. Indeed, shape-wise, these anions have the most complementary geometry to form H-bonds with the two N–H of the urea moiety. Once bound, the electron density of the anion delocalizes to the aromatic system of the receptor and thus causes repulsion between the double bond’s nitrogens. This thermal isomerization is prevented by the addition of acids such as TFA and triggered again when the media is basified.

**Figure 33:**
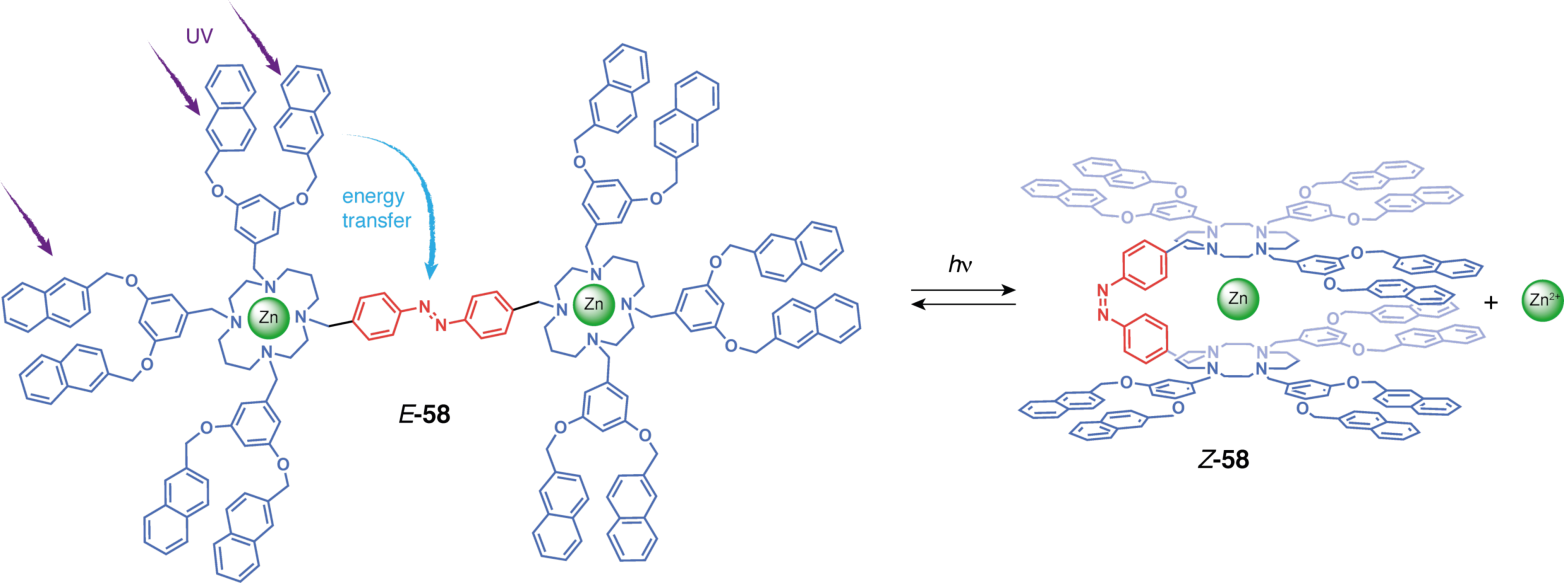
Cyclam-tethered tweezers and their different host–guest complexes depending on their configuration.

Ceroni et al. developed multiresponsive photoswitchable tweezers with cyclam moieties **58** [123]. The authors designed a dendrimer with a photoswitchable azobenzene moiety tethered to cyclam metal-coordinating units, which are linked to peripheral light-harvesting naphthyl moieties. Coordination of either Zn(II) or Cu(II) leads to the enhancement or impediment respectively of the photosensitization of the azobenzene photoisomerization. Titration with Zn(CF_3_SO_3_)_2_ indicated the formation of a 1:2 complex for *E-***58** with each cyclam binding to one zinc cation. However, the *cis*-configuration forms a 1:1 complex because the close proximity of the two cyclam units causes electrostatic repulsion between two Zn(II) (Figure 33). Similar coordination behavior was observed for copper(II). The emission of the naphthalene dendrons significantly decreased once incorporated in the uncomplexed tweezers, suggesting luminescence quenching from the azobenzene unit. Zn(II) coordination to the cyclam renders exciplex formation impossible, hence increasing drastically the energy transfer from naphthalene to azobenzene and providing an efficient sensitization of the photoisomerization. The photoswitching of *E-***58** to *Z-***58** thus enables a controlled Zn(II) release in solution due to the difference in binding affinity of the two forms. On the contrary, Cu(II) results in the absence of sensitized isomerization and a decreased isomerization quantum yield upon direct excitation of the azobenzene unit. These tweezers are thus a prime example of how a coordination stimulus can modulate the light-activated switching of the tweezers.

**Figure 34:**
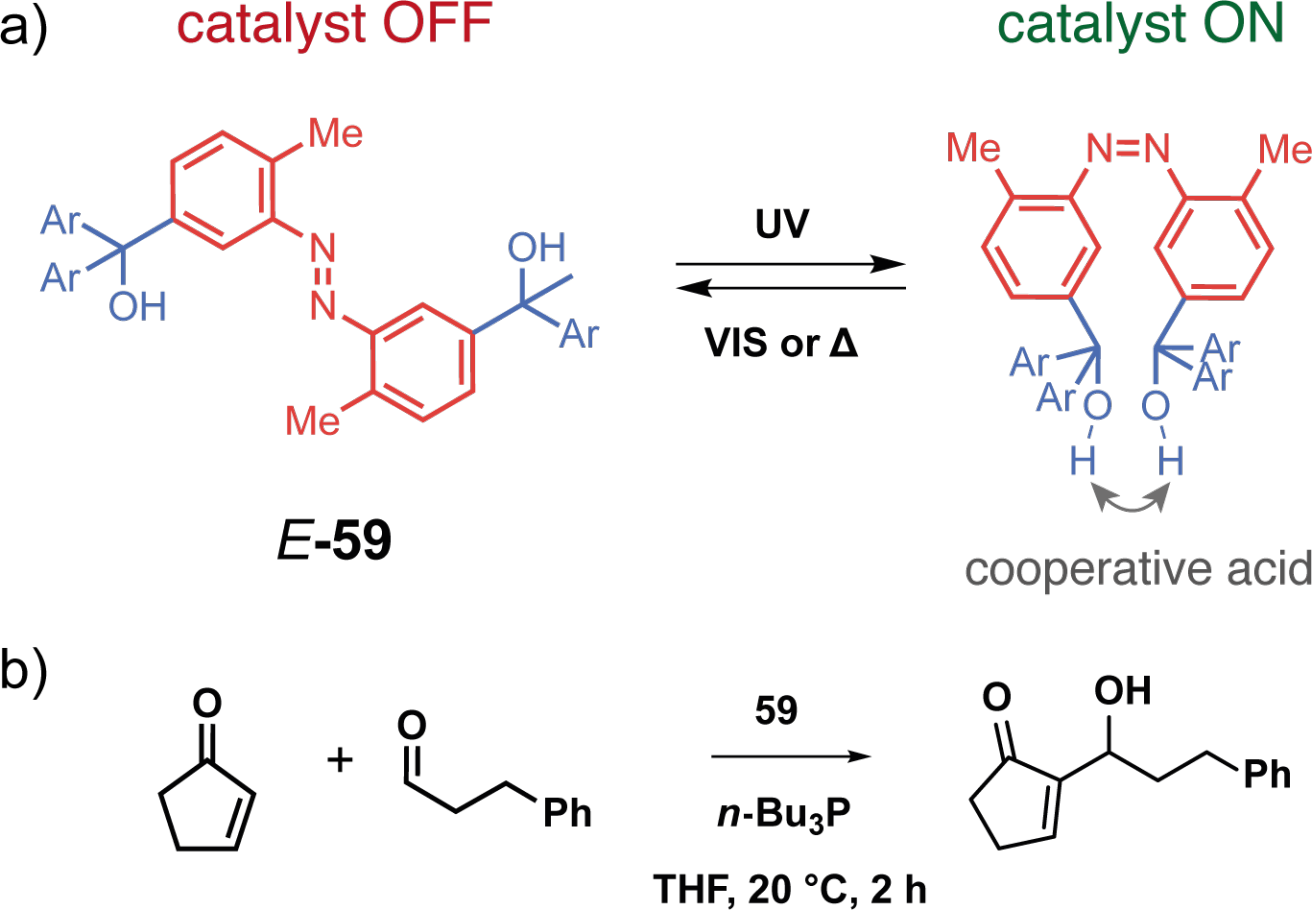
Azobenzene-based catalytic tweezers.

Beyond the photocontrol of substrate binding, photoswitchable tweezers are very attractive as switchable catalysts due to their precise spatiotemporal control but also for the lack of waste produced. In an early example, Imahori et al. developed an azobenzene-tethered bis-trityl alcohol **59** [124]. The tweezers in their closed *cis*-form, obtained after photoisomerization at 365 nm, act as a cooperative acid catalyst (Figure 34) due to the proximity of the two hydroxy groups. The intramolecular hydrogen bonding exacerbates the acidity of the alcohol groups and thus increases significantly the catalytic activity of the tweezers for the Morita–Baylis–Hillman reaction between the Michael acceptor 2-cyclopentenone and 3-phenylpropanal. Without a catalyst a conversion of around 27% is observed, whereas the addition of the *cis*-tweezers allowed 81% conversion. Irradiating the system with visible light (>420 nm) isomerizes the azobenzene to its *trans*-form and results in a much lower conversion of 38%. Thus, a drastic ON/OFF switching of the catalytic activity is achieved by **59**. The catalyst was further improved by the introduction of bulkier aryl groups to stabilize the *cis-*form or the addition of fluorinated aryls to improve consequently the cooperative effect between the two hydroxy groups.

**Figure 35:**
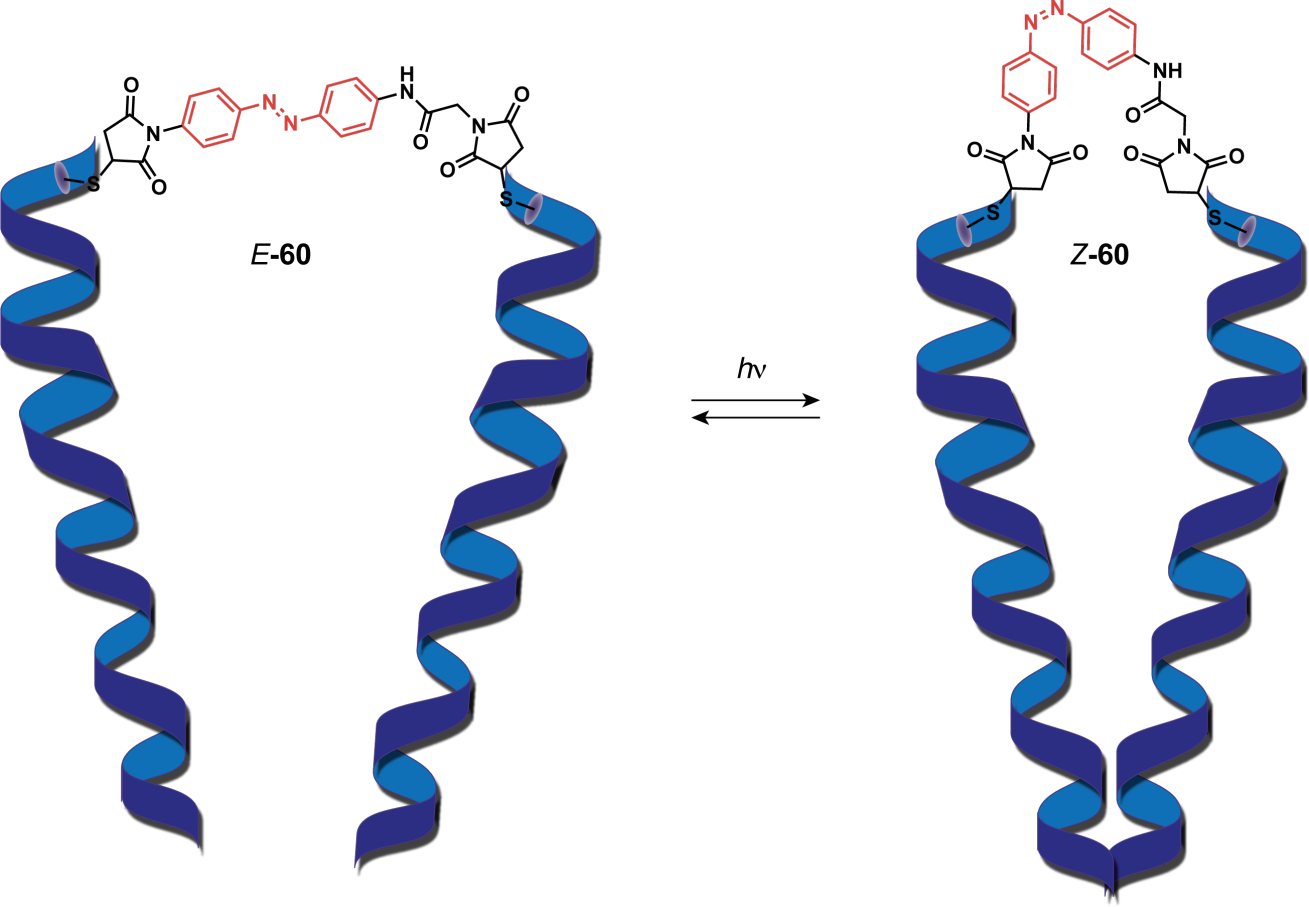
Photoswitchable PIEZO channel mimic.

At the interface with biological systems, Grutter et al. reported a photoswitchable PIEZO channel that mimics the mechanosensitive ion channels found in cell membranes [125]. These trimeric channels usually enable mechanotransduction, which is the process used by cells to convert mechanic signals into biochemical ones. For that purpose, the authors have tethered an azobenzene moiety to an engineered cysteine in the TM domain of the channel. This newly mutated channel was transfected afterward in HEK-P1KO cells, a cell line that commonly does not exhibit such channels. This resulted in the successful engineering of a light-gated ion channel **60** which opens up the trimer upon irradiation at 365 nm and closes it at 530 nm (Figure 35). Photoswitchable tweezers that act as ligand-gated ion channels as a means to investigate their mechanisms have been thoroughly discussed in recent reviews [126–128]. Most of these tweezers contain an azobenzene moiety as their switching unit, which is covalently bonded to protein residues through maleimide moieties. Their irradiation from *cis*-to-*trans* allows a distinct change in distance between the extremities of the molecule, which mimics the dynamic of ionotropic receptors.

#### Stilbene-based tweezers

The stilbene backbone consists of two phenyl rings connected by a central ethylene bridge. Possessing great photophysical properties, they have been used as models to investigate photochemical processes but also protein dynamics and biomembranes. *E*-Stilbene isomerizes with light to its hindered, and thus less-stable counterpart, *Z*-stilbene.

**Figure 36:**
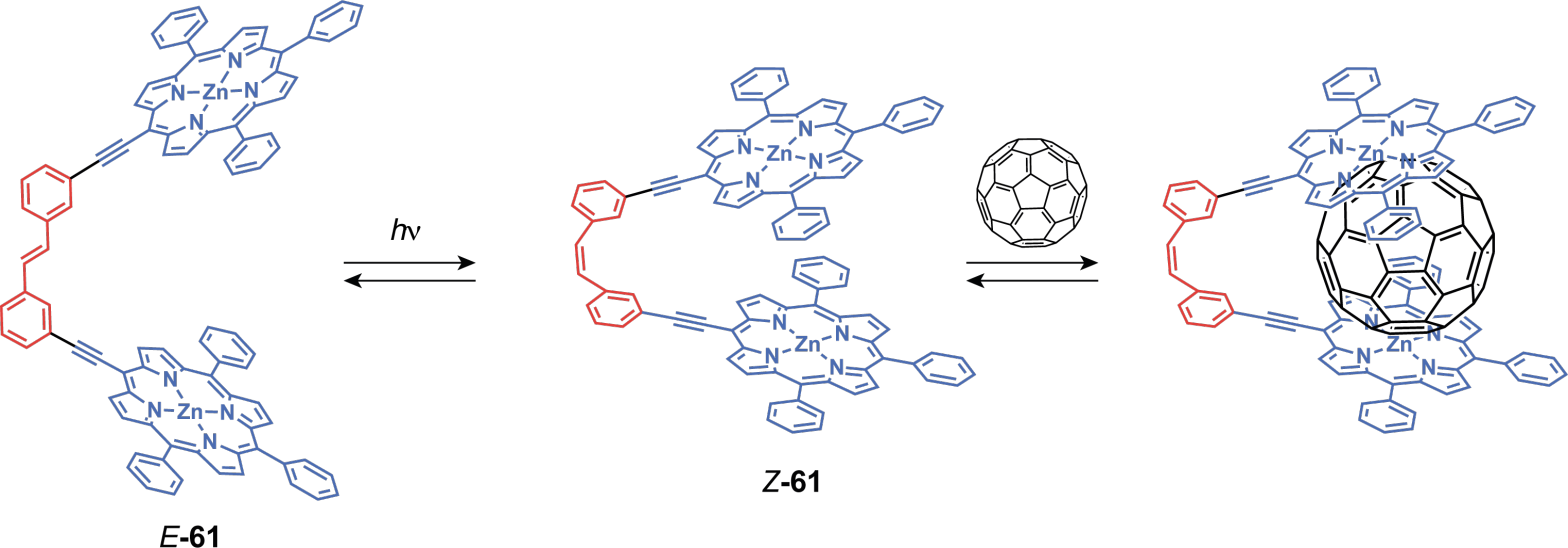
Stilbene-based porphyrin tweezers for fullerene recognition.

Jayawickramarajah et al. have reported stilbene tweezers containing two porphyrin arms for fullerene binding [129]. The *Z*-to-*E* photoisomerization of the double bond allows for the expansion of the cavity from 12 to 16 Å between the two Zn atoms (Figure 36). However, the wider cavity of *E*-**61** has exhibited less affinity to fullerenes. Indeed, the *Z*-isomer has the perfect bite size to accommodate the van der Waals diameters of C_60_ and C_70_ to form a 1:1 complex. Guest encapsulation induces a quenching of the porphyrin fluorescence via electron transfer allowing fluorescence titrations. *Z*-**61** presents high association constants (*K*_a_ = 2.9 ± 0.4 × 10^3^ M^−1^ for C_60_ and *K*_a_ = 8.2 ± 0.8 × 10^4^ M^−1^ for C_70_) while the *E*-isomer displays four- and five-times lower constants, respectively. Thus, these tweezers allow a photoregulated fullerene sequestration.

**Figure 37:**
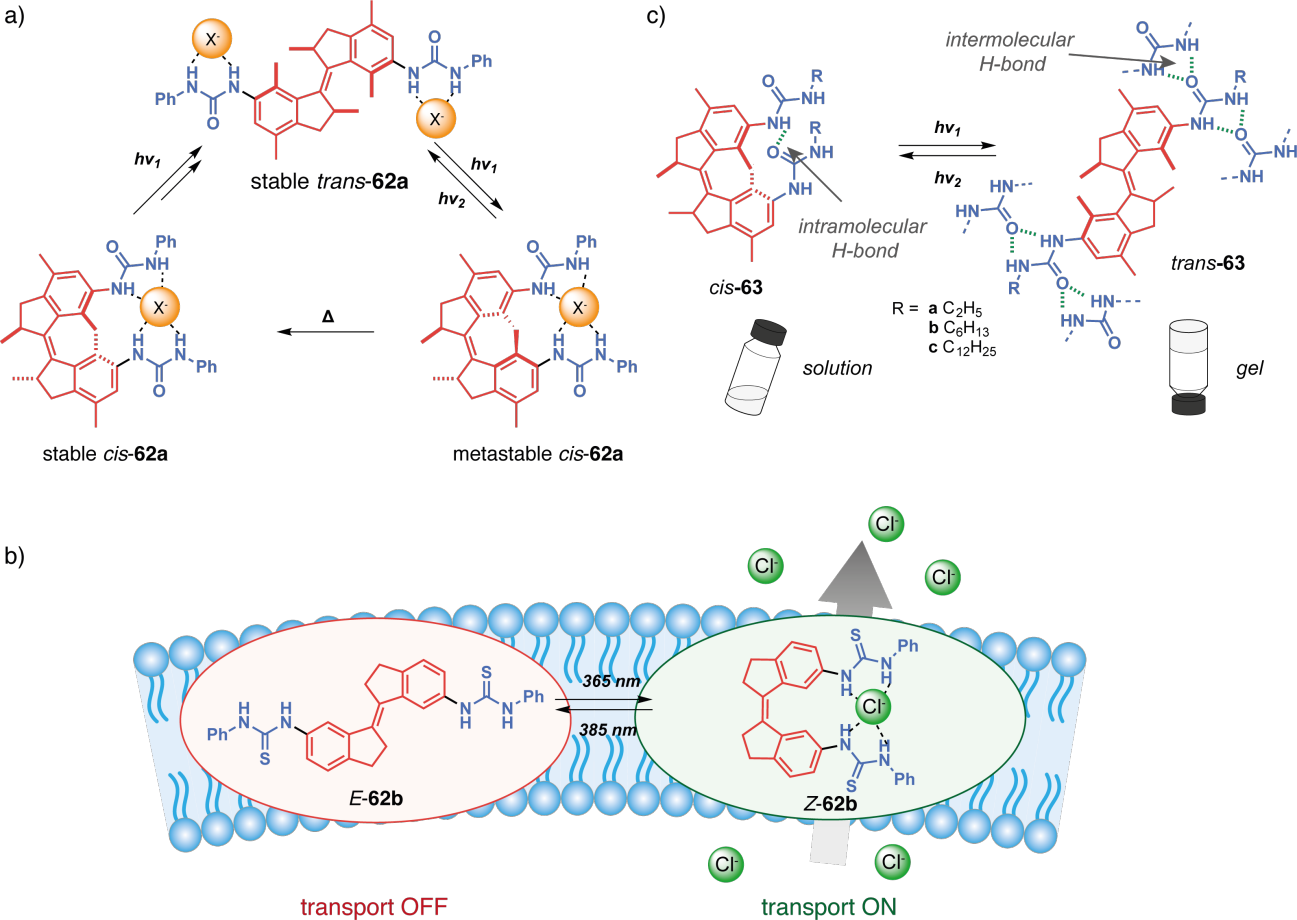
Stiff-stilbene-based tweezers with urea or thiourea functional units for a) anion binding, b) anion transport across membranes, and c) photocontrolled gelation.

Wezenberg in collaboration with Feringa reported an overcrowded bis-urea stiff-stilbene receptor highly selective of phosphates through H-bonding [130]. Its particularity stems from the difference in anion affinity of the three isomers that can be interconverted upon photoisomerization and thermal relaxation (Figure 37a). Stable *cis*-**62a** affords a mixture of metastable *cis* and stable *trans*-forms after applying a 312 nm irradiation. The *trans-***62a** isomerizes to the metastable *cis*-**62a** upon irradiation at 312 nm and reverts to itself at 365 nm. Upon heating, the metastable *cis* is completely converted into the stable *cis-*form. Titration experiments have shown that *trans*-**62a** forms a 1:2 complex with anions (such as Cl^−^, AcO^−^, H_2_PO_4_^−^) by H-bonds with the two urea moieties. On the other hand, the spatial proximity between the two urea for the *cis*-isomers allows a cooperative anion-binding via 4 hydrogen bonds and results in a 1:1 complex with much higher association constants and a high selectivity for H_2_PO_4_^−^. Interestingly, the metastable and stable *cis*-forms display significantly different affinities for H_2_PO_4_^−^ allowing a three-level control of the substrate binding. Later on, they designed less-hindered alkene-based bis-urea tweezers for anion binding by removing the methyl substituents on the cyclohexyl and phenyl moieties [131]. This small modification presents several advantages in comparison to the previously described system. The *E/Z* isomerization is achieved at a higher wavelength through 365/385 nm light irradiation which is more biocompatible, and the tweezers became stable in the presence of water. These attributes opened avenues for exploring their potential in trans-membrane transport.

Indeed, a few years later, Wezenberg and co-workers further developed an analog receptor **62b** with bis(thio)urea moieties in order to emulate anion-carrying membrane transport proteins [132]. The *Z*-isomer demonstrated a very high affinity for chloride, phosphate and acetate anions. Photoswitching to the *E-*form induced a much lower binding affinity demonstrating the interest of these systems in light-gated regulation of transmembrane anion transport. Subsequent liposomal membrane transport assays revealed significantly enhanced transport activities of the *Z*-tweezers for chloride, exhibiting at least a sevenfold increase when compared to their *E* counterparts. Starting from *E*-**62b**, in situ light-induced chloride transport was subsequently demonstrated, showcasing excellent efficiency relative to pure *Z*-**62b.** Notably, even with prolonged irradiation, no degradation of the lipid bilayer membrane was observed following exposure to UV light. The predominantly electrogenic transport mechanism allowed light-gated membrane depolarization marking a pioneering example of a fully artificial switch capable of generating an in situ chloride gradient. More recently, Wezenberg et al. demonstrated that transport activity and binding affinity are not necessarily correlated using bis(amidopyrrols)-functionalized stiff-stilbene with dimethyl-substituted cyclohexyl [133]. While both isomers exhibited weak binding affinities for chloride due to steric hindrance of the dimethyl groups, the *Z*-configuration showed much higher in situ transport activities than *E*-tweezers. All of these results demonstrate the potential of photoactive tweezers to develop molecular pumps analogous to transmembrane proteins with potential applications as physiological tools or therapeutic agents.

Concomitantly, Wezenberg in collaboration with Beves reported a novel application for switchable urea-based anion receptors to modulate the size of hydrogen-bonded phosphate oligomers [134]. The authors demonstrated by DOSY NMR that hydrogen phosphates form oligomers via anti-electrostatic hydrogen bonding in DMSO. Such oligomers can bind in an anionic way to the urea moieties of the tweezers leading to larger assemblies. Indeed, the *E*-isomer presents two divergent urea binding sites that allow cross-linked assemblies of approximately three times the size. The photoswitching to *Z*-isomers that are no longer able to bridge oligomers causes a significant increase in the diffusion coefficient associated with a decrease in the size of the assemblies. This system thus allows the control of diffusion and could prove to be very efficient in regulating the transport of small molecular species. Song et al. also reported similar bis-urea tweezers based on a stiff-stilbene photoswitchable moiety [135]. The photoisomerization from the *E*-configuration to the *Z* one allows for the complexation of anions such as phosphate and carboxylate through hydrogen bonding. Though binding constants for the latter have proven to be a bit lower than the former. These guests release is triggered upon irradiation of distinct light. This system targets pharmaceutical applications such as drug delivery and channeling anions across a cell membrane, as cytotoxicity tests showed negligible toxicity of the tweezers at concentrations lesser than 20 μM.

Going beyond guest binding, Wezenberg and Feringa reached photocontrolled gelation properties using molecular tweezers. They developed a reversible gel-to-sol transition using an overcrowded alkene-based bis-urea organogelator **63** [136]. The *trans*-form enables intermolecular hydrogen bonds resulting in the formation of a fibrous network and efficient gelation in various aromatic hydrocarbon solvents at low concentrations (as low as 0.4 mg^.^mL^−1^). In contrast, the *cis*-form presents intramolecular H-bonding that prevents the formation of extended assemblies and gelation of the solvent. Thus, the UV irradiation (312 nm) of hexyl-substituted *trans-***63b** in toluene triggers the complete transition from the gel state to the sol one in under 15 min. Furthermore, irradiating at 365 nm with gentle heating induces the gelation of the solution demonstrating the reversibility of the transition and control of macroscopic properties. This concept was further expanded to control supramolecular polymerization and also induce sol–gel transition with similar photoresponsive stiff-stilbene bis-urea monomers [137]. Again, the *cis*-monomer is inactive due to the intramolecular hydrogen bonds. Upon photoisomerization, it is converted to an active *trans*-isomer that enables the self-assembly process with intermolecular H-bonding. The photoinduced supramolecular polymerization is cooperative and under kinetic control in toluene, creating polymers with controlled gelation properties.

**Figure 38:**
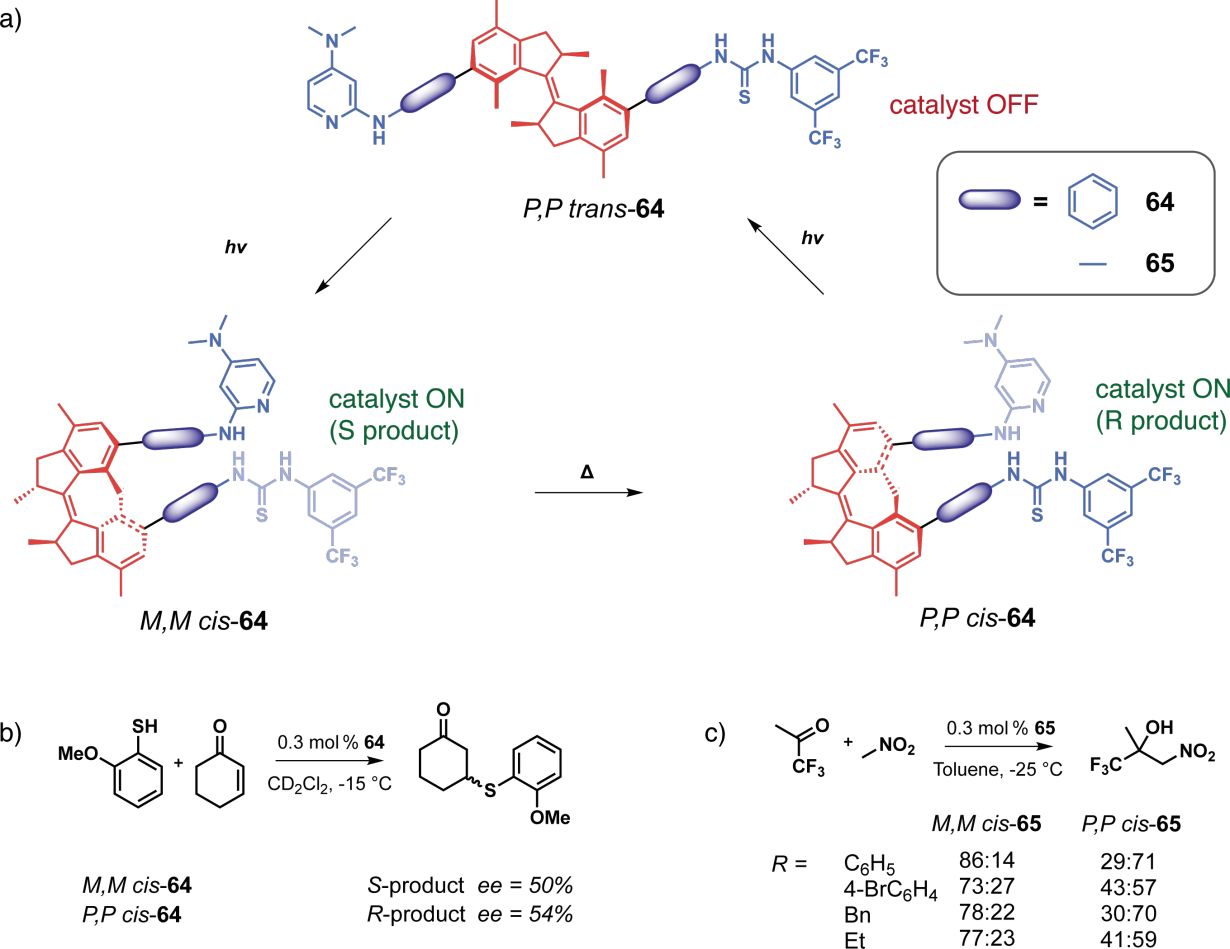
Feringa’s photoswitchable organocatalyst (a) and different catalyzed reactions with that system (b).

Feringa et al. took stiff-stilbene-based tweezers a step further in catalysis by developing a photoswitchable asymmetric catalyst to control the chiral space of their catalyst by photoswitching [138]. They designed tweezers **64** that integrates around an overcrowded chiral stiff-stilbene core a DMAP moiety on one side and of a thiourea donor group on the other one. Like previous systems, the tweezers can be converted between three states upon irradiation, from an open *trans*-state to a closed *cis*-*M*-helicity and after thermal helix inversion to a *cis*-*P*-helicity (Figure 38a). In both *cis*-forms, the two moieties are in proximity and function cooperatively as organocatalysts for the Michael addition reaction between cyclohexanone and thiophenol. Indeed, for this reaction, the thiourea moiety activates the Michael acceptor through hydrogen bonding whereas the thiol substrate’s nucleophilicity is enhanced with the assistance of DMAP. A remarkable control of both the activity and enantioselectivity of the reaction was achieved: from a low activity of the *trans*-form to a high activity of the *cis*-form associated with opposite enantioselectivity between (*P*,*P*)-*trans* and (*M*,*M*)-*cis*-isomers. The group developed a new generation to induce enantioselectivity in the Henry reaction (Figure 38b) [139]. This system works like the one previously described. It is, however, much more efficient as a result of a more constrained chiral pocket. This is caused by the removal of the phenyl spacers, which inherently increases the cooperativity of the two organocatalysts thanks to the proximity. Thus, great conversion and stereoselectivity have been observed for the (*M*,*M*)-*cis*-isomer of this catalyst. A dynamic control of chirality in phosphine ligands was also achieved using the same concept and was applied in enantioselective catalysis for Pd-catalyzed desymmetrization reaction [140].

#### Thioindigo and hemithioindigo-based tweezers

Thioindigos, derived from the well-known indigo dye, are a particular class of photochromic switches composed of a central photoisomerizable double bond linked to two thioindigo moieties [141]. A key characteristic of these photochromes is their ability to undergo reversible isomerization upon exposure to visible light, achieved through a 180° rotation around the central double bond. The metastable isomers typically exhibit enhanced thermal stability, and both forms display high rigidity and planarity. Hemithioindigos (HTI) are unsymmetrical compounds composed of a stilbene part that is linked to a thioindigo moiety. They have been explored as photoswitches these past few years for their excellent chromophoric properties [142]. The *Z*-to-*E* switching of hemithioindigos changes significantly the electronic properties of the system and allows the use of visible light instead of UV. However, the thermal reversion from the metastable *E*-isomer to the thermodynamic *Z* one possesses a significantly higher barrier than azobenzene. That property renders them powerful bistable systems.

**Figure 39:**
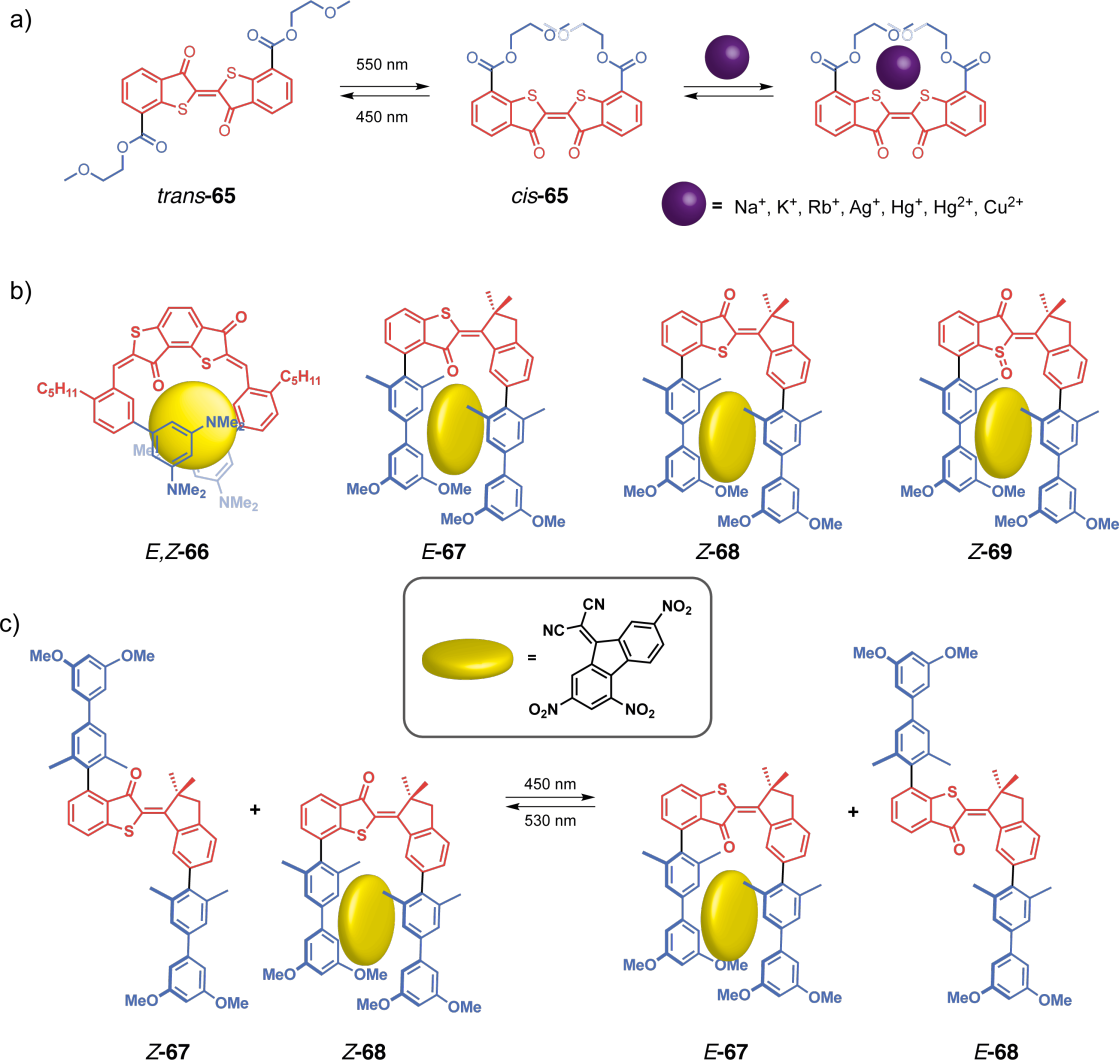
a) Irie and Takeshita’s thioindigo-based molecular tweezers. b) Family of hemithioindigo-based molecular tweezers in their closed conformation developed by Dube. c) Mechanism of photoswitching of HTI tweezers and example of guest.

Irie and co-workers in a seminal work introduced photoresponsive molecular tweezers based on the thioindigo derivative **65** containing ethylenedioxy chains for the photoextraction of metal cations [143]. The stable *trans-*configuration displays a rodlike structure with the two ethylenedioxy chains apart from each other and no binding affinity for metal cations. Photoisomerization of *trans*-**65** by 550 nm visible light to *cis-***65** brings the two chains in proximity and allows the formation of a cavity similar to that of a crown ether (Figure 39a). The photogenerated *cis-*form was able to successfully extract hard alkaline cations (Na^+^ < Rb^+^ < K^+^) and even soft metal cations such as Ag^+^, Hg^2+^, and Cu^2+^. Indeed, the *cis-*configuration creates a cavity with four oxygen and two sulfur donor atoms thanks to the cooperativity between the two ethylenedioxy, thus accommodating the metal cations. The authors have successfully transported silver cations with this system across a 1,2-dichloroethane membrane from one aqueous phase to another upon photoisomerization at 529 nm. This study showed early results of photoregulated cation transport across a liquid membrane.

Dube et al. designed a photoswitchable bis-hemithioindigo receptor **66** that can bind electron-poor aromatic guests once folded (Figure 39b) [144]. The planar and more thermodynamically stable *Z,Z*-configuration shifts selectively and with high conversion to the helical *E,Z* one upon irradiation at 420 nm. Steric hindrance of the two terminal phenyls generates the helicity of the structure and creates a hydrophobic pocket. The latter can then bind guests such as 9-dicyanomethylene-2,7-dinitrofluorene through polar aromatic interactions. This *E,Z-*form reversibly and quantitatively reverts to the *Z,Z*-isomer with heat and thus effectively allows guest release.

The authors then reported an HTI multiresponsive system that can reversibly modulate its affinity to electron-deficient aromatic substrates by applying irradiation [145]. Two pairs of tweezers **67** and **66**, both in *Z*-configuration, have been designed where, by default, one is in its closed form (**68**) and the other in its open form (**67**). The closed tweezers can intercalate a 9-dicyanomethylene-2,7-dinitrofluorene guest whereas the open one has no interaction with it (Figure 39c). The mixture of the two tweezers can be simultaneously isomerized with visible light to their respective *E*-isomer, shifting the guest from the previous closed *Z*-**68** system to the newly closed tweezers *E*-**67**. Different molecular recognition patterns can hence be exploited through the same stimulus. Building on the same tweezers design, Dube et al. introduced a more polar sulfoxide hemithioindigo in tweezers **69** to attain enhanced photoswitching properties [146]. Incorporating this function improves significantly the thermal stability of the *E*-isomer and allows full reversibility of the switching. The energy barrier between the two isomers is over 33 kcal·mol^−1^, granting bistability to the system. The authors observed an increase in binding constants as the sulfoxide function offers the *Z*-isomer an additional H-bonding to the guest. Switching between two bistable states with irradiation therefore offers ON/OFF control of aromatic substrate binding.

#### Diarylethene-based tweezers

Diarylethenes is a prominent class of photochromic molecules discovered by Irie and colleagues in the 1980s that undergo light-induced reversible cyclization [147]. Their widespread adoption is attributed to exceptional photochromic properties, high quantum yields, and remarkable fatigue resistance. The open and closed forms exhibit distinct electronic properties and rigidities. Indeed, the open form exists in parallel and antiparallel conformations, rapidly interconverting at room temperature. While only the antiparallel conformation undergoes photocyclization, the parallel form possesses optimal geometry for cooperative substrate binding. However, the absence of substantial geometrical changes upon photocyclization limits their appeal for the design of switchable molecular tweezers and only limited examples have been reported.

**Figure 40:**
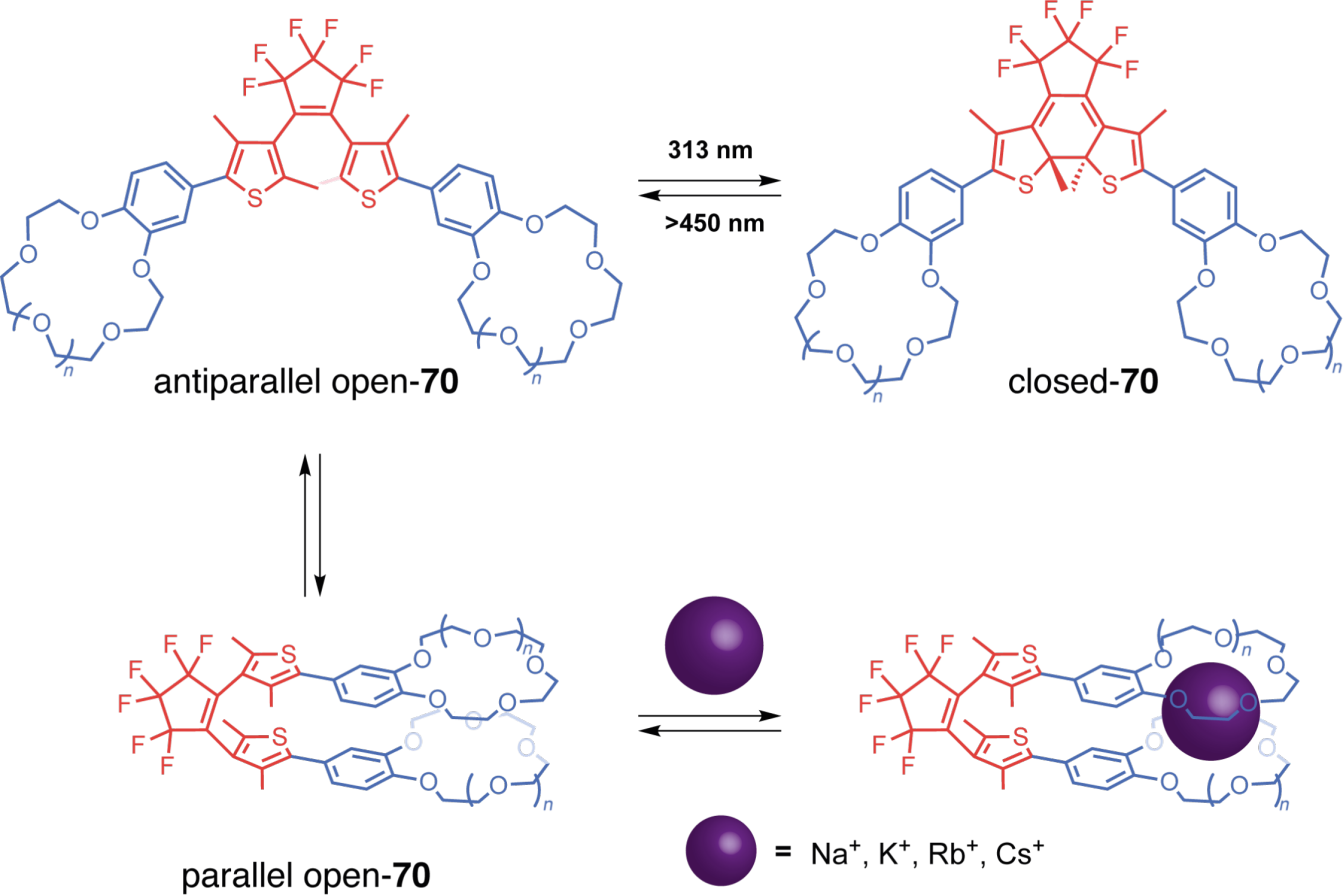
Dithienylethylene crown ether-bearing molecular tweezers reported by Irie and co-workers.

In 1996, Irie et al. reported a tweezer-like receptor consisting of bis(benzothienyl)ethene with two boronic acid moieties, capable of modulating its affinity for glucose upon photoirradiation [148]. Circular dichroism analysis revealed that glucose recognition occurs in the parallel conformation of the tweezers in a 1:1 fashion as each boronic acid site forms esters with the saccharide’s hydroxy groups. Upon conversion to the closed-ring form by photoirradiation at 313 nm, the boronic acid groups become separated, impeding the formation of the complex and resulting in the release of the guest. Irie and Takeshita subsequently reported similar tweezers based on dithienylethene and diarylethene structures, incorporating crown ether moieties for the recognition of metal cations (Figure 40) [149–150]. An almost quantitative photoswitching was observed for tweezers **70** after irradiating at 313 nm with a 9:91 open/closed ratio at the photostationary state (PSS). The energy barrier between parallel and antiparallel conformations of the open form was estimated at 67 kJ·mol^−1^, demonstrating fast equilibrium at room temperature. The parallel conformation of open-**70** allows for the cooperative binding of alkali metal ions, forming a 1:1 sandwich-like complex with the two crown ether moieties. Metal ion extraction studies from aqueous phases were conducted for Na^+^, K^+^, Rb^+^, and Cs^+^, revealing an increase in cation extractability with an increase in cation size. Reversible cation uptake was achieved upon photocyclization, as the closed-ring form released the ions. This study highlights the potential of molecular tweezers for photocontrolled metal cation binding.

All of these systems demonstrate the diversity of structures and versatility of photoswitchable molecular tweezers and their wide range of potential applications.

## Conclusion

In conclusion, the emergence of switchable molecular tweezers in the last two decades has marked a significant milestone at the intersection of supramolecular chemistry and the emerging field of molecular machines. This combination has led to significant advances, characterized by the relentless pursuit of switching units capable of responding to a wide range of stimuli, from chemical to electrochemical or photochemical, to achieve reversible operation of these tweezers. The large conformational change induced by the mechanical motion, resulting in at least two different states with different geometries, has not only facilitated the development of recognition systems with pronounced ON/OFF responses but has also stimulated the exploration of other applications. In particular, applications in areas such as allosteric catalysis, biology, smart materials, and multiresponsive systems have emerged and hold great promises. The biological applications where a small artificial molecule can regulate biological processes in a biorthogonal way are a great application of switchable tweezers. In the same domain, the control of the transport of ions or small molecules across membranes could have a big impact in particular if the system can be designed to go up-gradient and develop molecular pumps analogs to transmembrane proteins but on a much smaller scale. The characteristics of switchable molecular tweezers allow them to play a key role in such an active transport mechanism.

From a broader perspective, these switchable molecular tweezers should play a key role in the design of future functional molecular machines. A current challenge is the integration of molecular tweezers into more sophisticated molecular machines and making each component interact with each other, which would mirror the intricate communication systems observed in biological systems. In addition, there is an urgent need to overcome the limitations of binary switches by venturing into the domain of multidimensional systems. Switchable molecular tweezers appear as a great candidate to meet this challenge. The future path requires the merging of diverse properties within a single system, orchestrating a synergy that promises unparalleled prospects in the field of supramolecular chemistry.

## Data Availability

Data sharing is not applicable as no new data was generated or analyzed in this study.
